# Supplementary material for: Synthesis and Biological Activity of Glycosyl Thiazolyl Disulfides Based on Thiacarpine, an Analogue of the Cytotoxic Alkaloid Polycarpine from the Ascidian Polycarpa aurata
Source: Mar Drugs. 2025 Mar 9;23(3):117. doi: 10.3390/md23030117 (PMC11943723; doi:10.3390/md23030117)
Supplement: Supplementary file 1 [file marinedrugs-23-00117-s001.zip › marinedrugs-3498863-supplementary.pdf]

Supplementary Material:

Synthesis and Biological Activity of Glycosyl Thiazolyl Disulfides Based on Thiacarpine, an Analogue of the Cytotoxic Alkaloid Polycarpine from the Ascidian *Polycarpa aurata*

Dmitry N. Pelageev [1,] *, Yuri E. Sabutski [1], Svetlana M. Kovach [2], Nadezhda N. Balaneva [1], Ekaterina S. Menchinskaya [1], Ekaterina A. Chingizova [1], Anna L. Burylova [1,3] and Victor Ph. Anufriev [1]

[1] G. B. Elyakov Pacific Institute of Bioorganic Chemistry, Russian Academy of Sciences, Prospect 100 let Vladivostoku 159, 690022, Vladivostok, Russian Federation
[2] Institute of High Technologies and Advanced Materials, Far Eastern Federal University, Ajax Bay 10, Russky Island, 690922, Vladivostok, Russian Federation
[3] Institute of the World Ocean, Far Eastern Federal University, Ajax Bay 10, Russky Island, 690922, Vladivostok, Russian Federation

The copies of 1H NMR and 13C NMR spectra for compounds **4a-j, 5a-j**

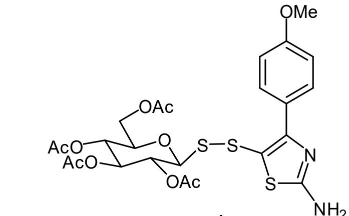

4a

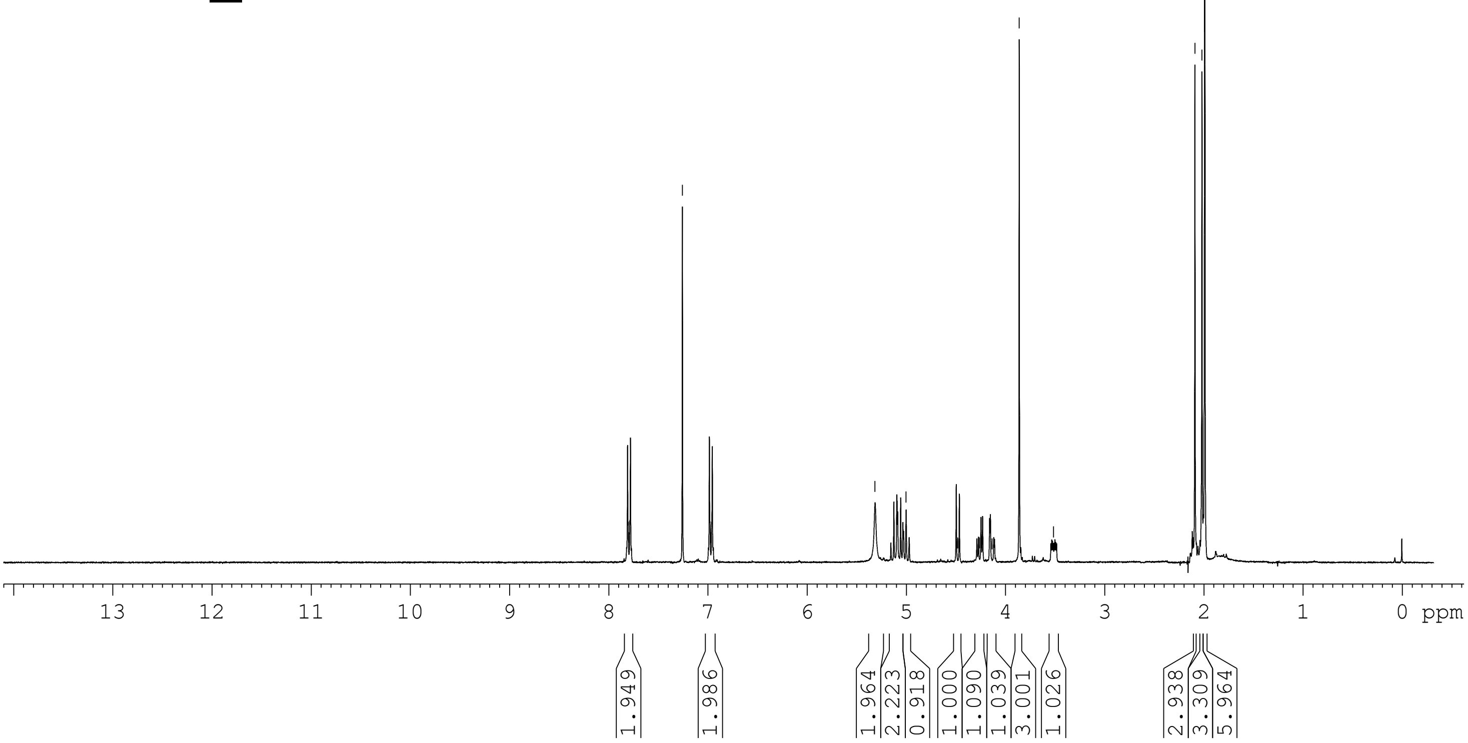

7.799
7.260
6.974
5.318
5.094
5.006
4.483
4.263
4.138
3.864
3.518
2.092
2.022
1.993
13 12 11 10 9 8 7 6 5 4 3 2 1 0 ppm
1.949
1.986
1.964
2.223
0.918
1.000
1.090
1.039
3.001
1.026
2.938
3.309
5.964

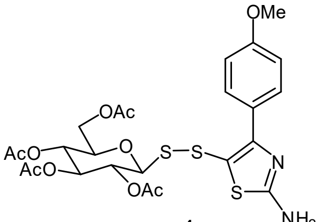

4a

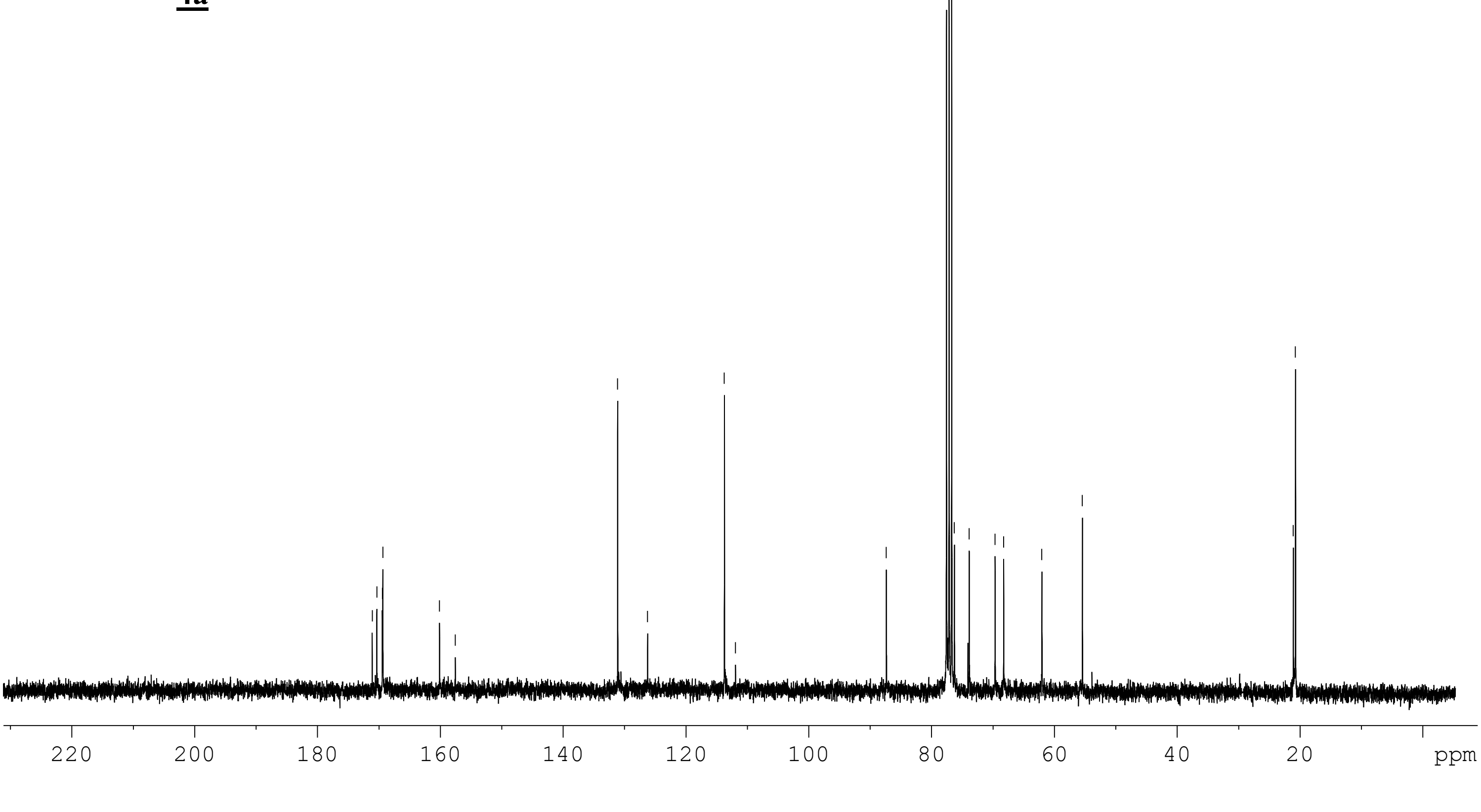

171.075
170.323
169.430
169.337
160.142
157.574
131.132
126.250
113.748
111.943
87.382
77.160
76.285
73.878
69.666
68.268
62.036
55.436
21.093
20.742
220 200 180 160 140 120 100 80 60 40 20 ppm

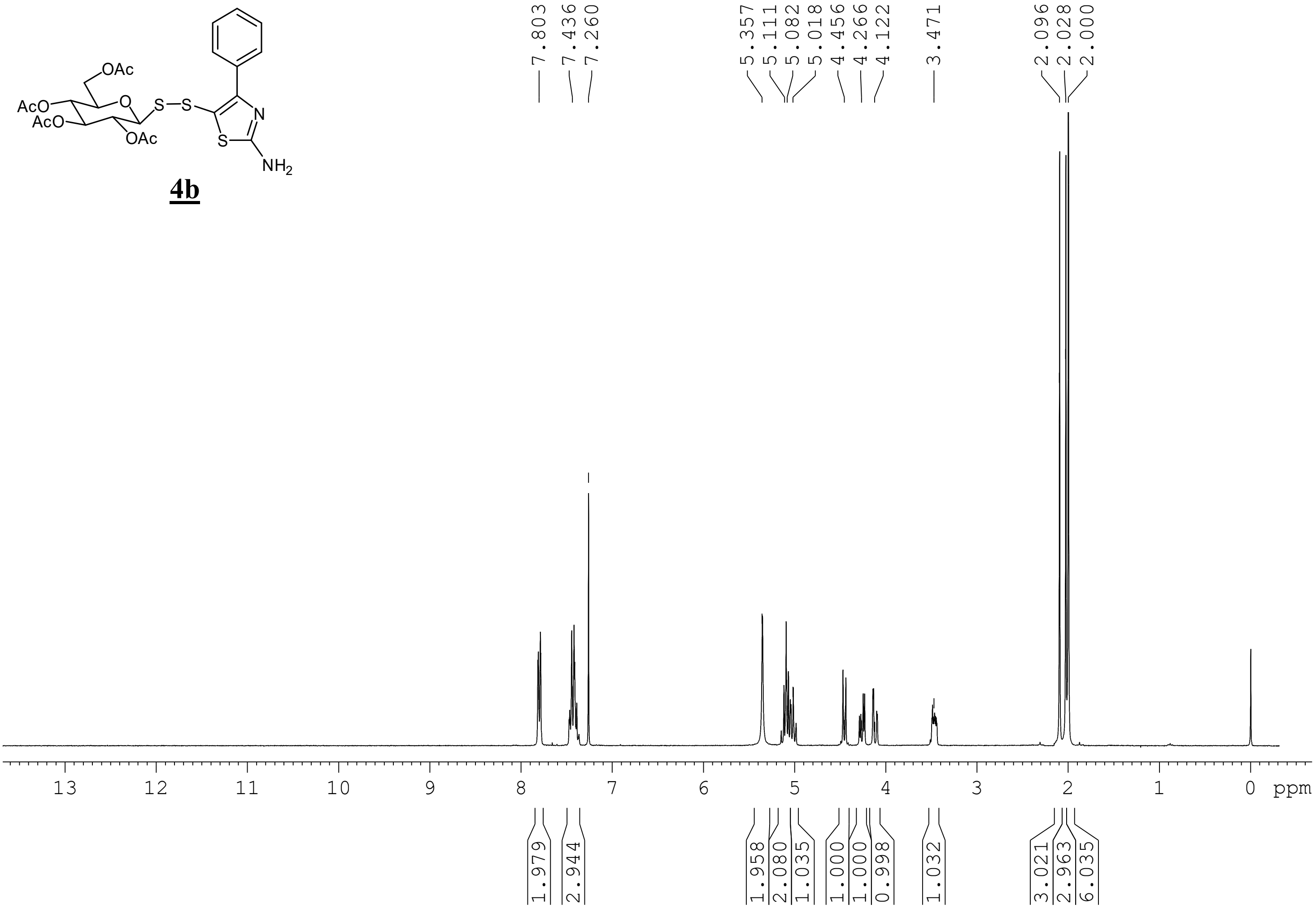

OAc
AcO
AcO
OAc
O
S–S
S
N
NH2
4b
7.803
7.436
7.260
5.357
5.111
5.082
5.018
4.456
4.266
4.122
3.471
2.096
2.028
2.000
13 12 11 10 9 8 7 6 5 4 3 2 1 0 ppm
1.979
2.944
1.958
2.080
1.035
1.000
1.000
0.998
1.032
3.021
2.963
6.035

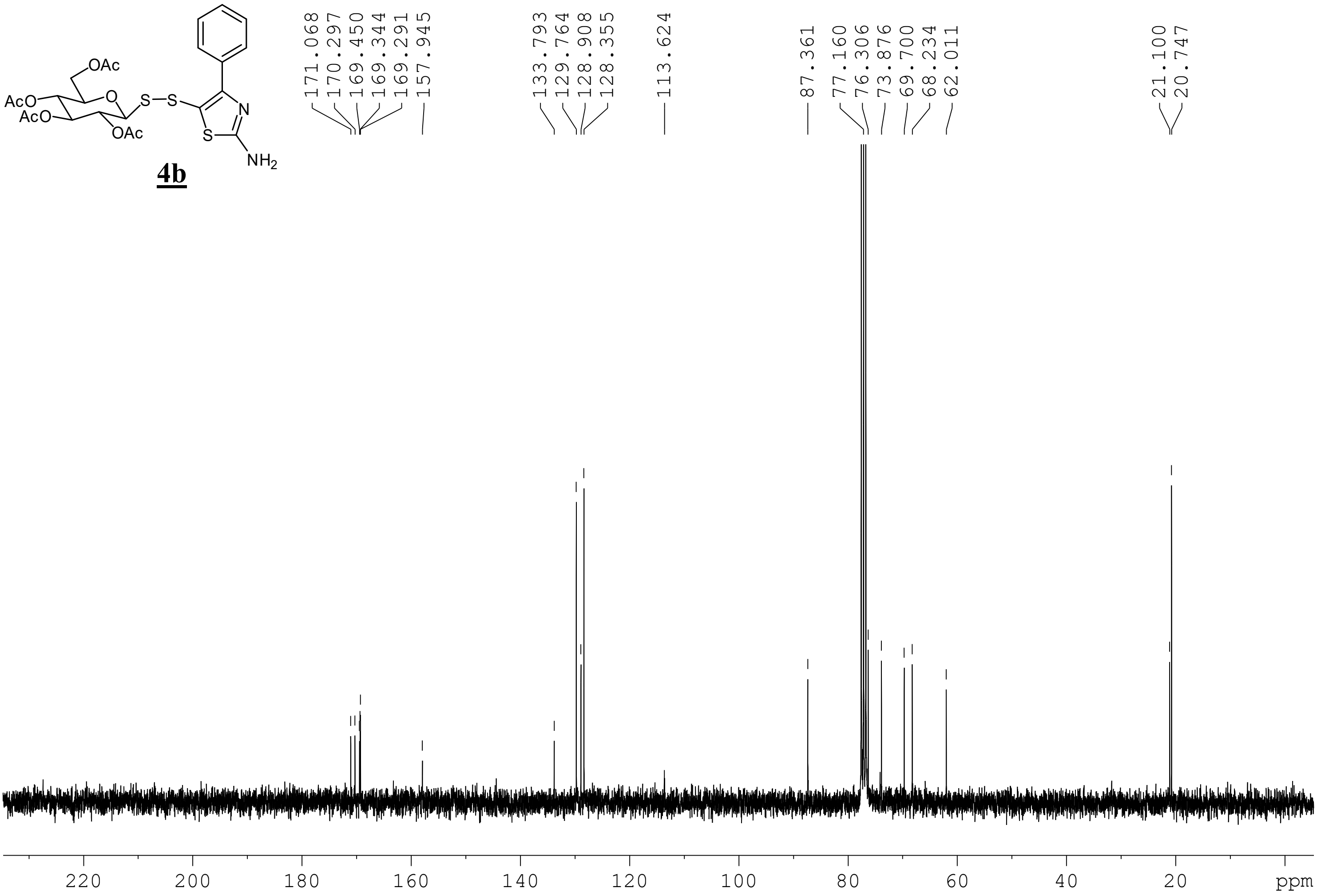

OAc
AcO
AcO
OAc
O
S–S
S
N
NH2
4b
171.068
170.297
169.450
169.344
169.291
157.945
133.793
129.764
128.908
128.355
113.624
87.361
77.160
76.306
73.876
69.700
68.234
62.011
21.100
20.747
220 200 180 160 140 120 100 80 60 40 20 ppm

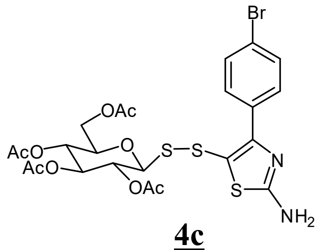
Br
OAc
AcO
AcO
O
S–S
N
S
OAc
NH2

4c

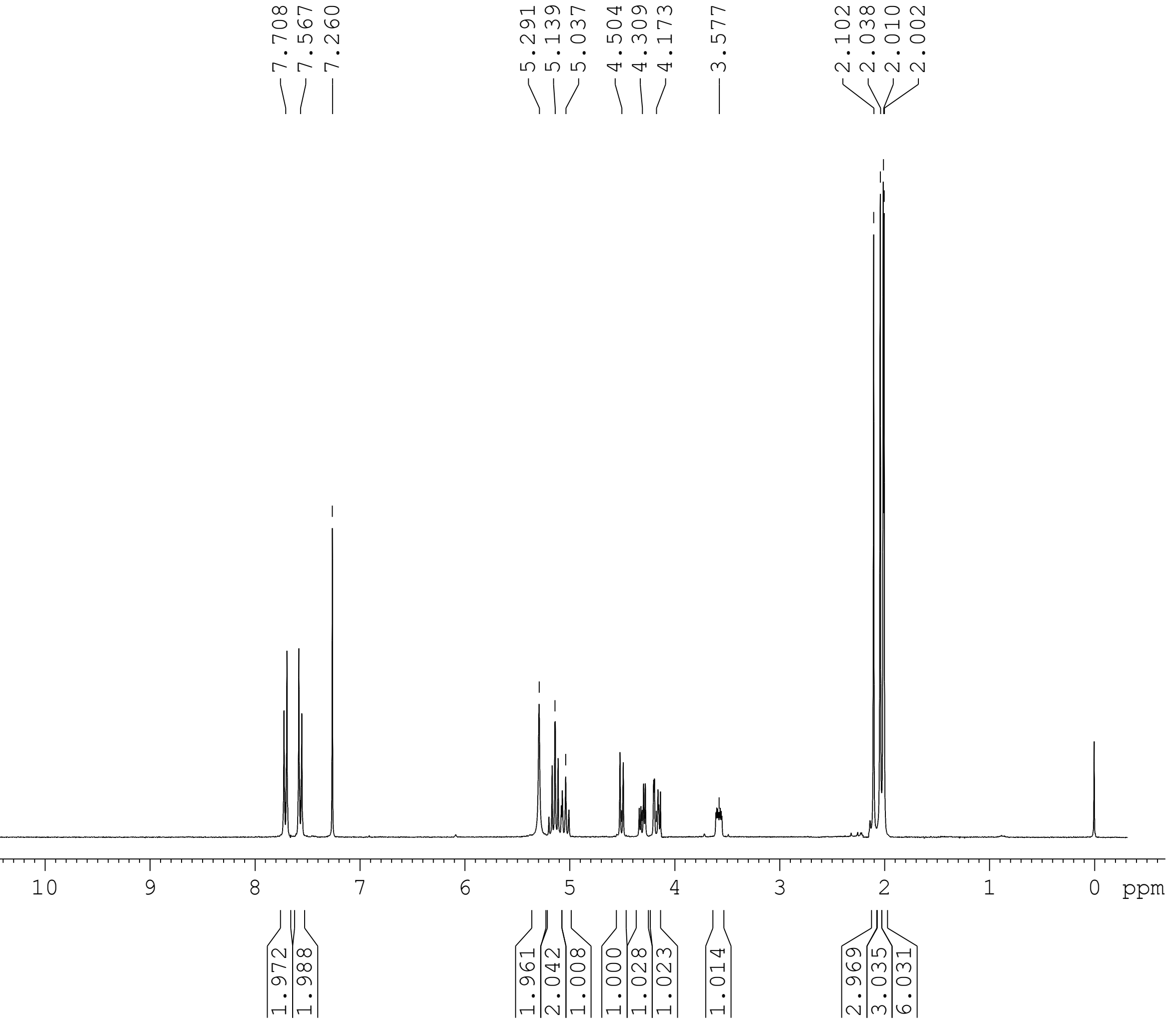
7.708
7.567
7.260
5.291
5.139
5.037
4.504
4.309
4.173
3.577
2.102
2.038
2.010
2.002
13 12 11 10 9 8 7 6 5 4 3 2 1 0 ppm
1.972
1.988
1.961
2.042
1.008
1.000
1.028
1.023
1.014
2.969
3.035
6.031

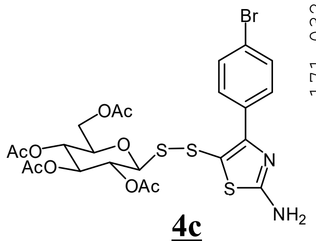
Br
OAc
AcO
AcO
O
S–S
N
S
OAc
NH2

4c

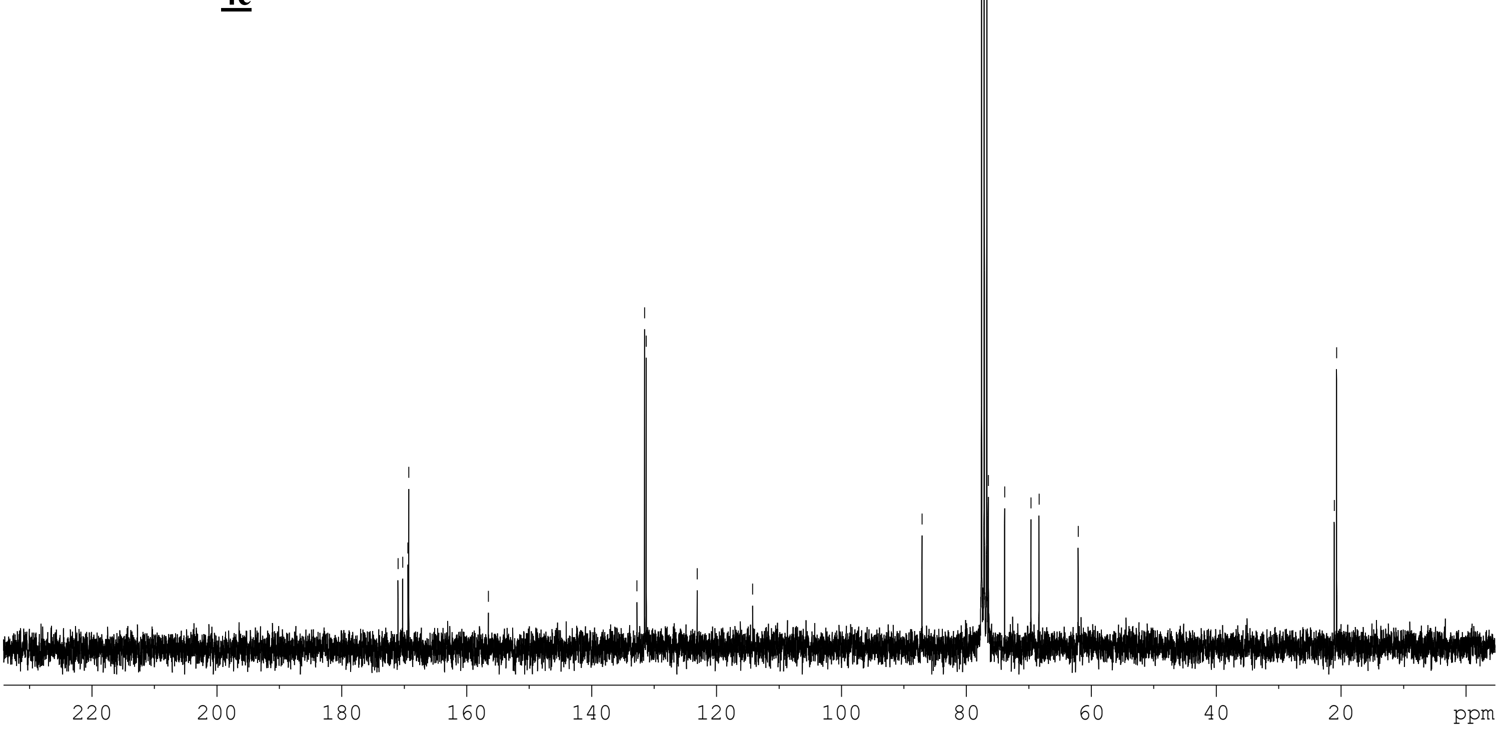
171.032
170.285
169.438
169.298
156.564
132.756
131.544
131.279
123.125
114.232
87.117
77.160
76.482
73.890
69.669
68.389
62.120
21.112
20.739
220 200 180 160 140 120 100 80 60 40 20 ppm

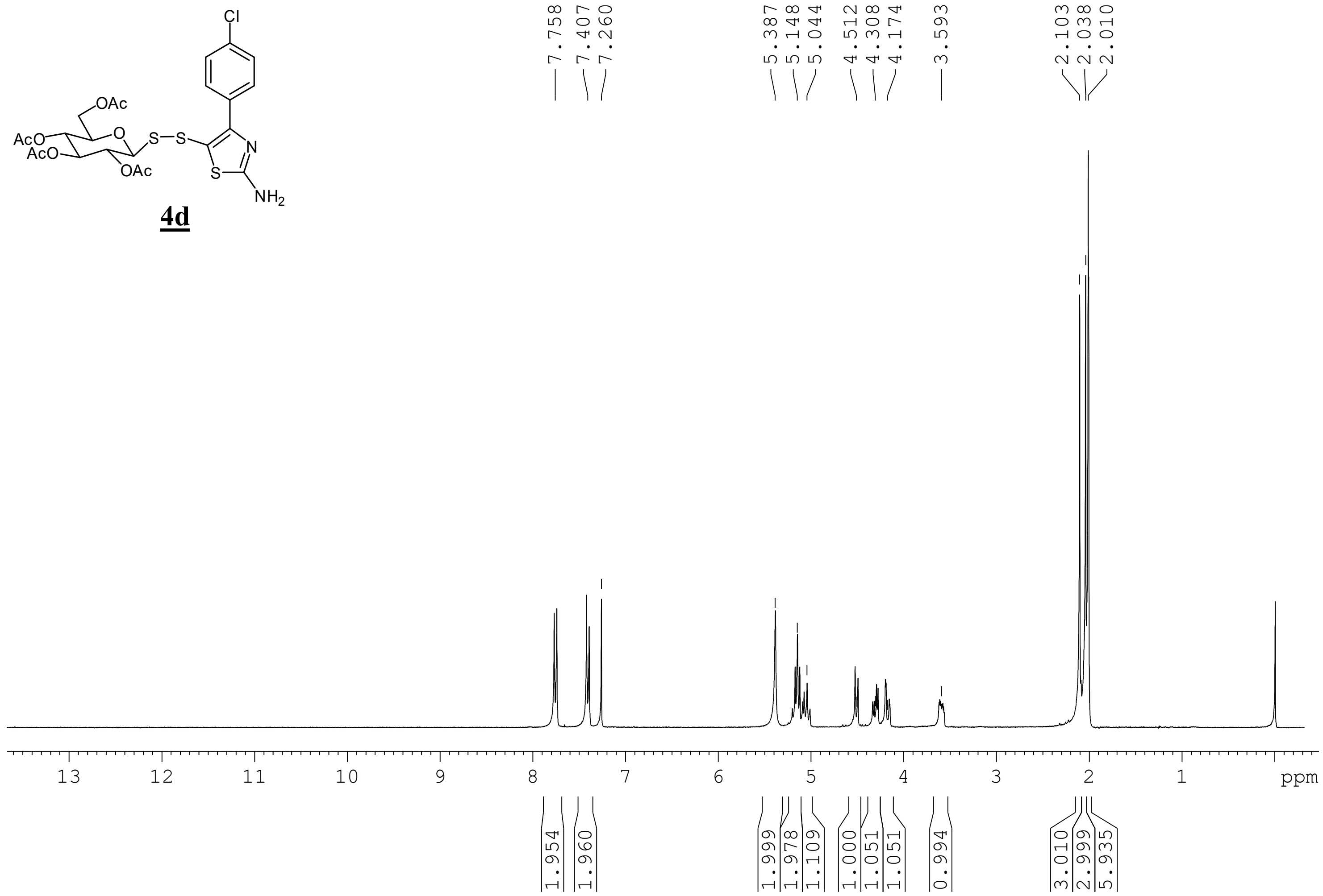

4d
Cl
OAc
AcO
AcO
OAc
S–S
N
S
NH2
7.758
7.407
7.260
5.387
5.148
5.044
4.512
4.308
4.174
3.593
2.103
2.038
2.010
13 12 11 10 9 8 7 6 5 4 3 2 1 ppm
1.954
1.960
1.999
1.978
1.109
1.000
1.051
1.051
0.994
3.010
2.999
5.935

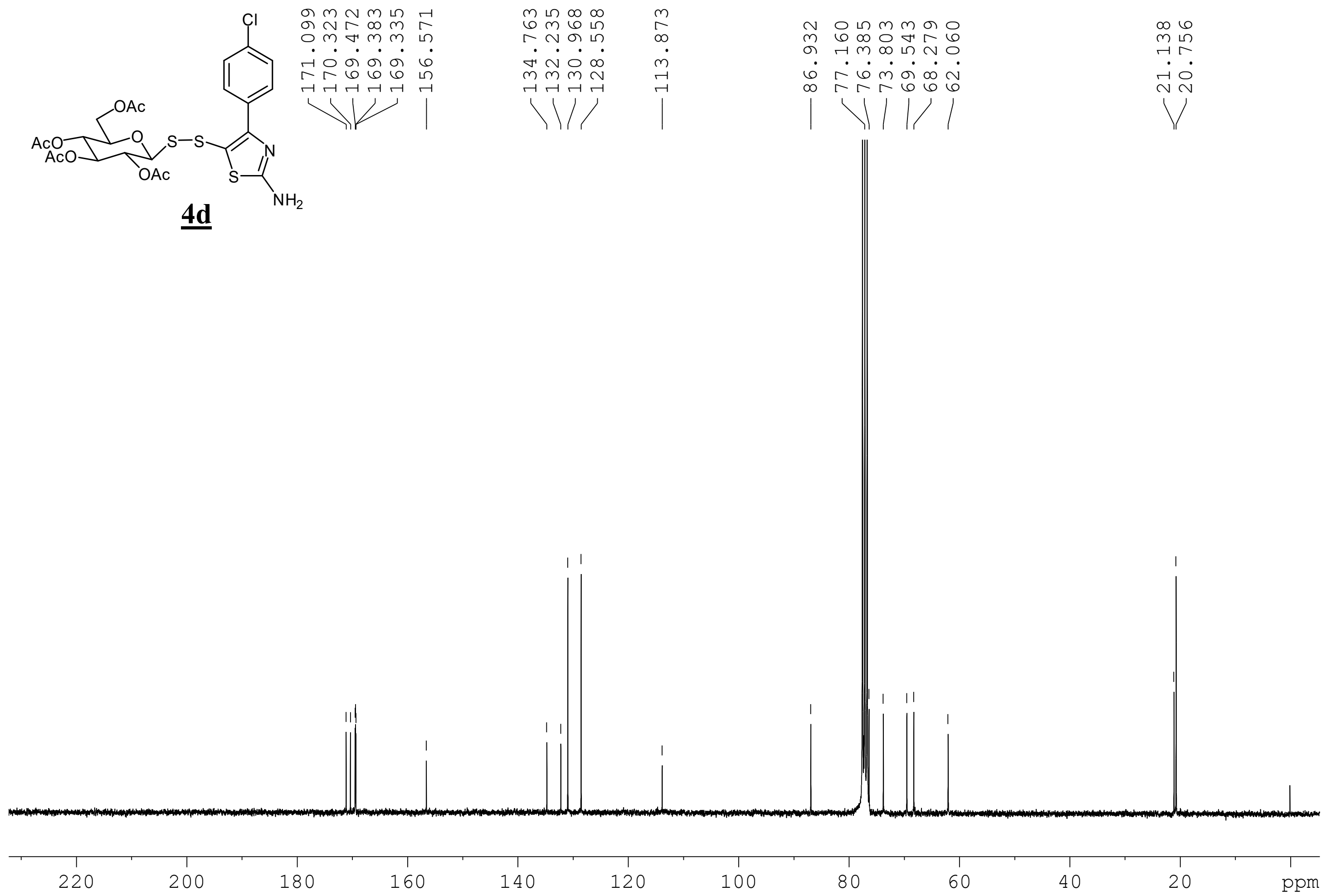

4d
171.099
170.323
169.472
169.383
169.335
156.571
134.763
132.235
130.968
128.558
113.873
86.932
77.160
76.385
73.803
69.543
68.279
62.060
21.138
20.756
220 200 180 160 140 120 100 80 60 40 20 ppm

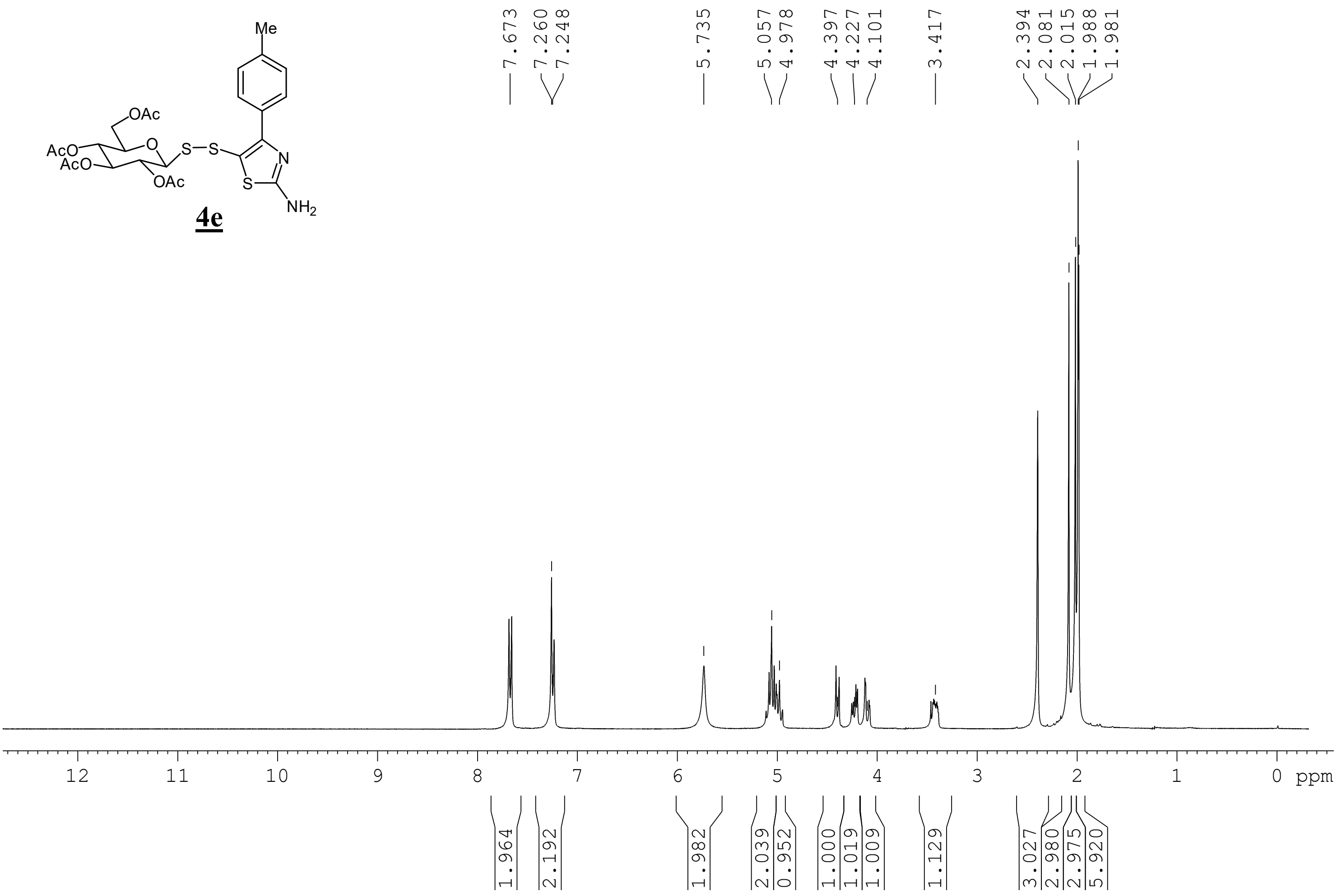
Me
OAc
AcO
AcO
O
OAc
S–S
N
S
NH2
4e
7.673
7.260
7.248
5.735
5.057
4.978
4.397
4.227
4.101
3.417
2.394
2.081
2.015
1.988
1.981
12 11 10 9 8 7 6 5 4 3 2 1 0 ppm
1.964
2.192
1.982
2.039
0.952
1.000
1.019
1.009
1.129
3.027
2.980
2.975
5.920

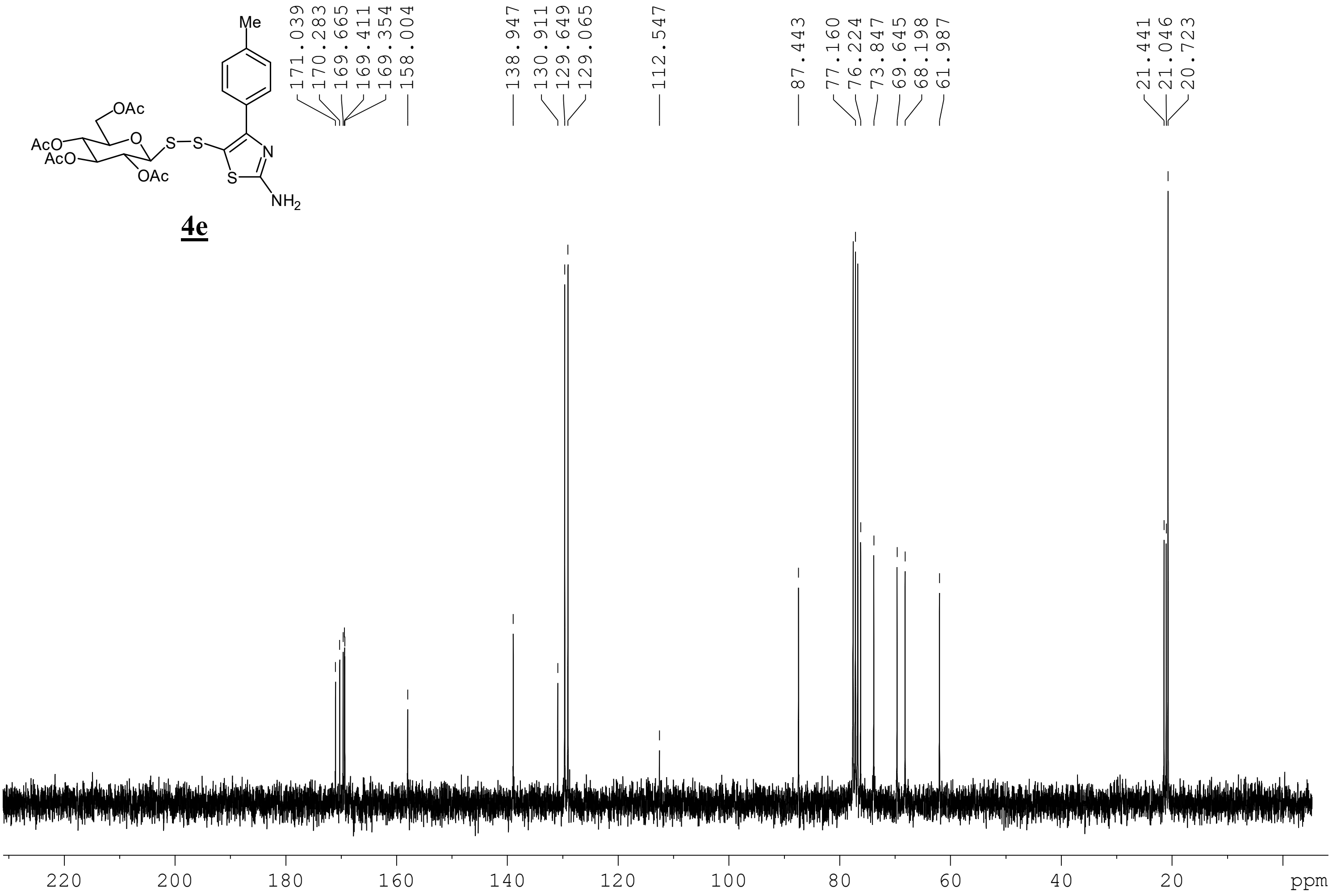
Me
OAc
AcO
AcO
O
OAc
S–S
N
S
NH2
4e
171.039
170.283
169.665
169.411
169.354
158.004
138.947
130.911
129.649
129.065
112.547
87.443
77.160
76.224
73.847
69.645
68.198
61.987
21.441
21.046
20.723
220 200 180 160 140 120 100 80 60 40 20 ppm

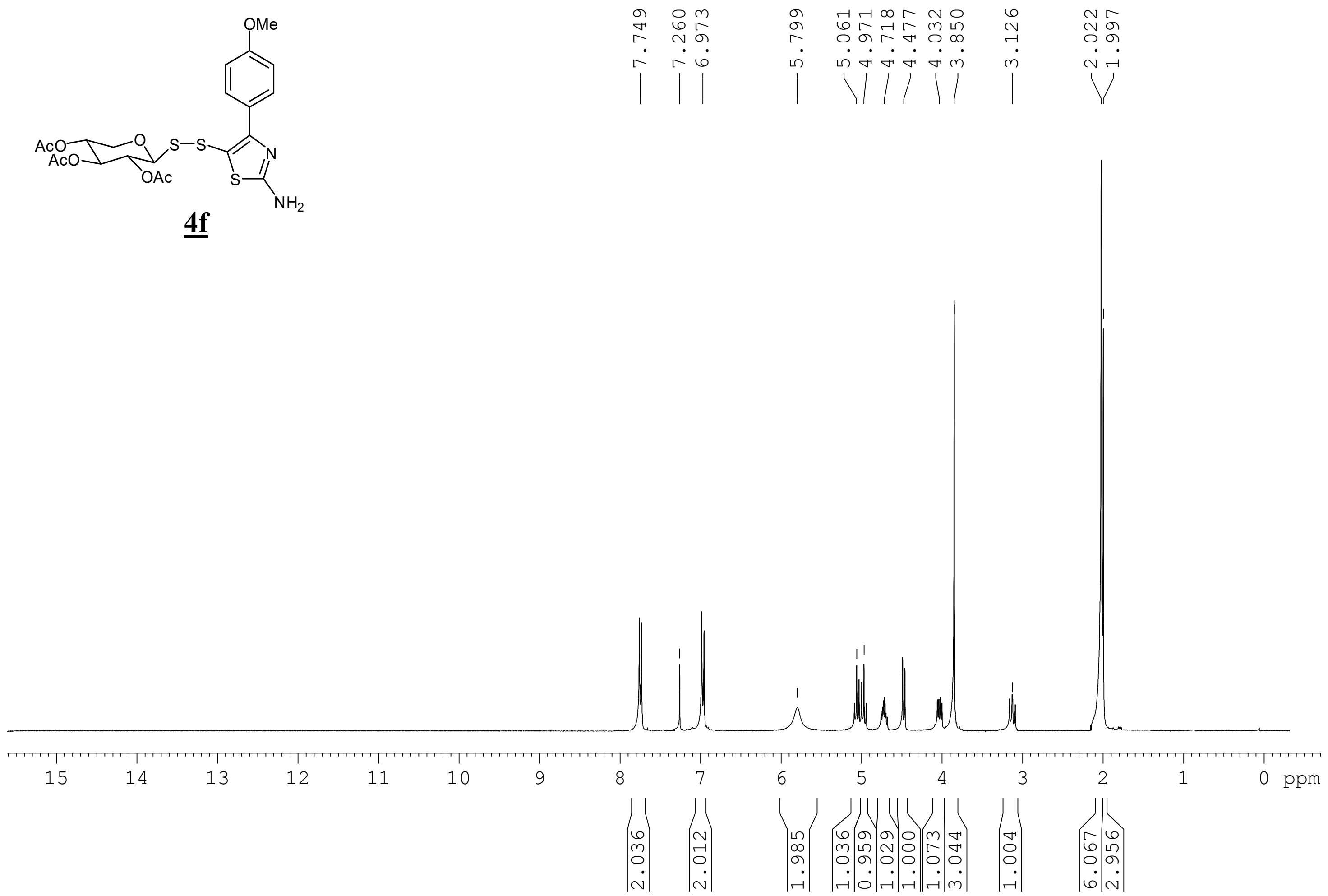
OMe
AcO
AcO
O
S—S
N
OAc
S
NH2
4f
7.749
7.260
6.973
5.799
5.061
4.971
4.718
4.477
4.032
3.850
3.126
2.022
1.997
15 14 13 12 11 10 9 8 7 6 5 4 3 2 1 0 ppm
2.036
2.012
1.985
1.036
0.959
1.029
1.000
1.073
3.044
1.004
6.067
2.956

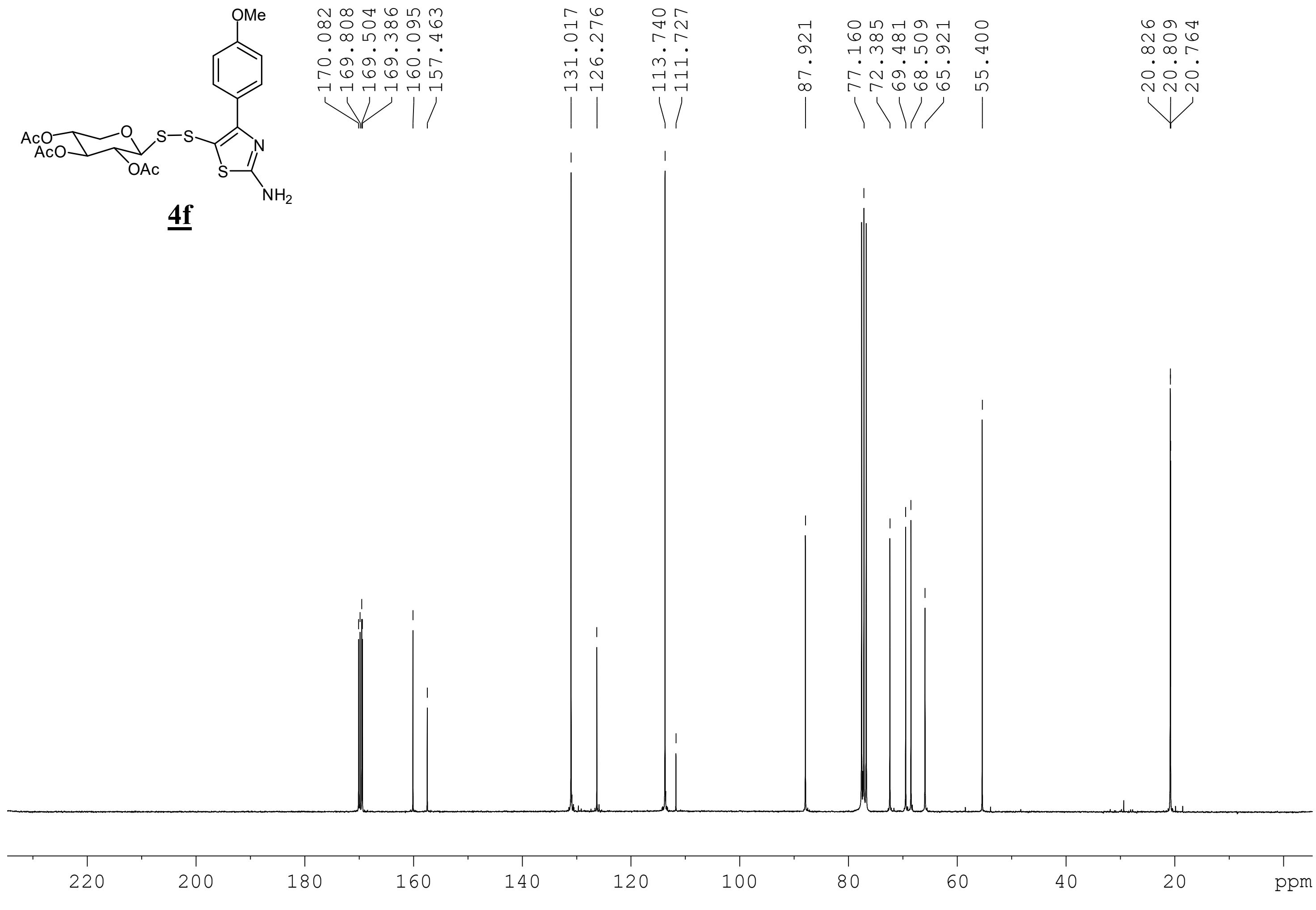
OMe
AcO
AcO
O
S—S
N
OAc
S
NH2
4f
170.082
169.808
169.504
169.386
160.095
157.463
131.017
126.276
113.740
111.727
87.921
77.160
72.385
69.481
68.509
65.921
55.400
20.826
20.809
20.764
220 200 180 160 140 120 100 80 60 40 20 ppm

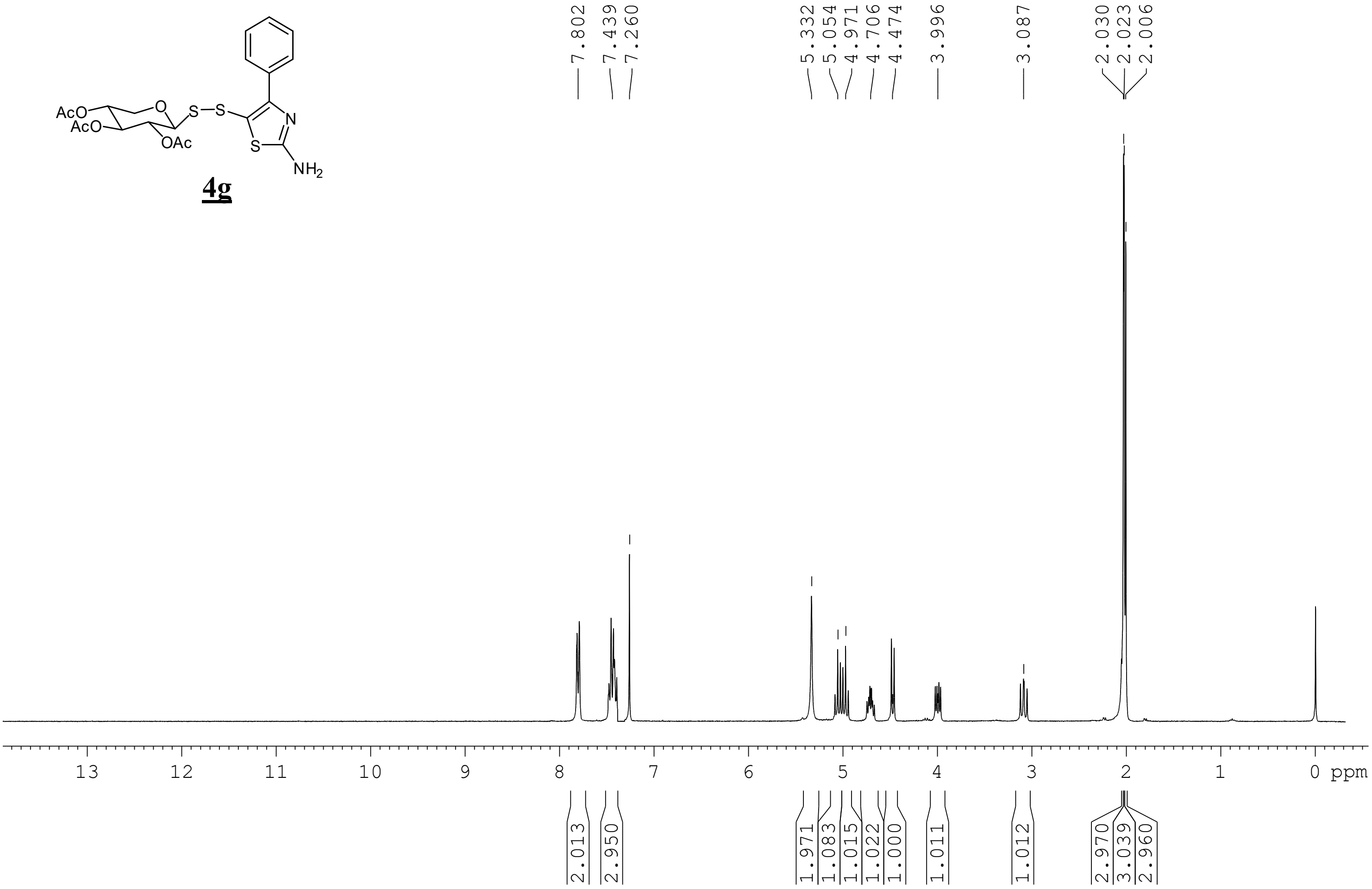
AcO
AcO
O
OAc
S–S
S
N
NH2
7.802
7.439
7.260
5.332
5.054
4.971
4.706
4.474
3.996
3.087
2.030
2.023
2.006
13 12 11 10 9 8 7 6 5 4 3 2 1 0 ppm
2.013
2.950
1.971
1.083
1.015
1.022
1.000
1.011
1.012
2.970
3.039
2.960

4g

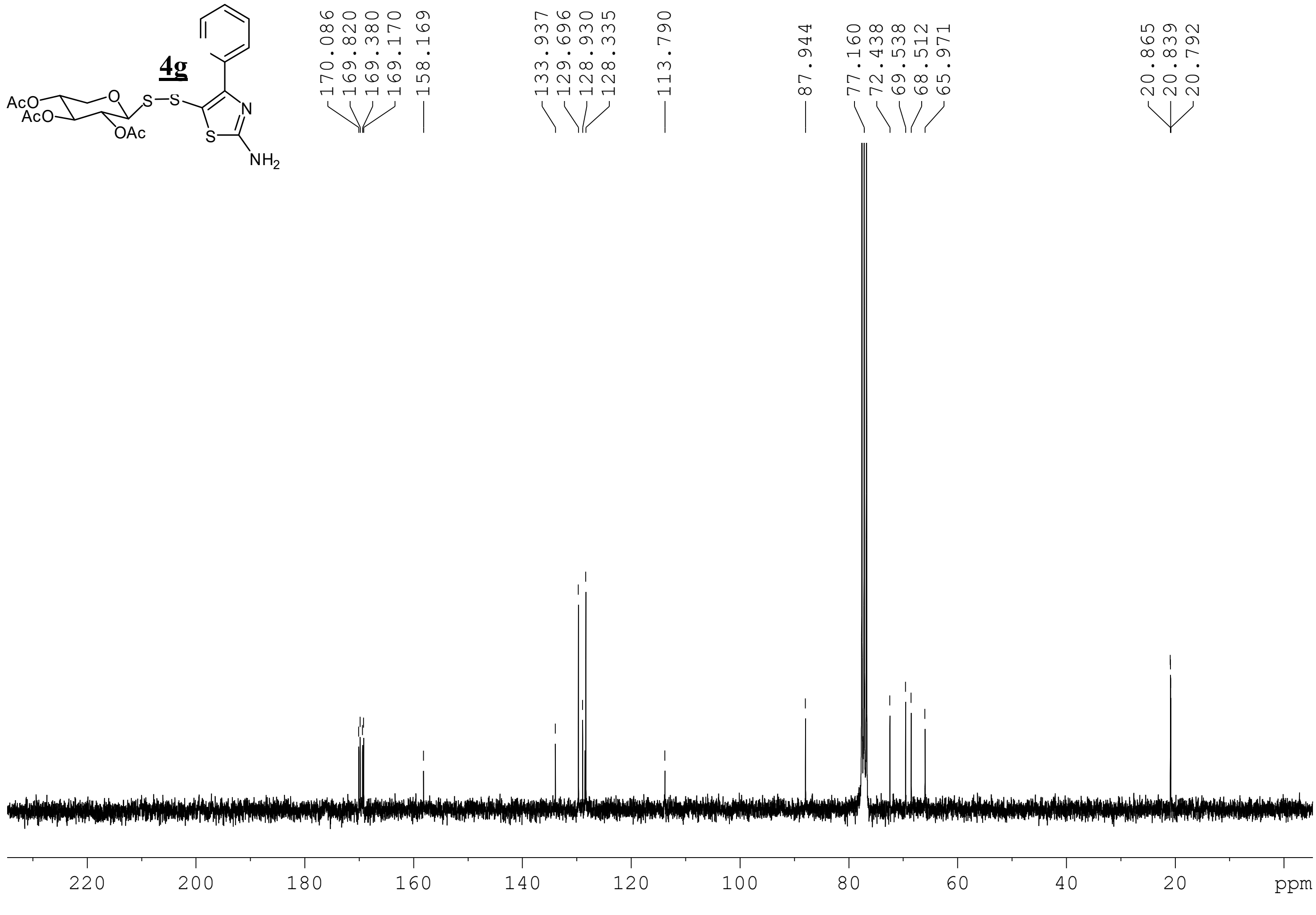
AcO
AcO
O
OAc
S–S
S
N
NH2
170.086
169.820
169.380
169.170
158.169
133.937
129.696
128.930
128.335
113.790
87.944
77.160
72.438
69.538
68.512
65.971
20.865
20.839
20.792
220 200 180 160 140 120 100 80 60 40 20 ppm

4g

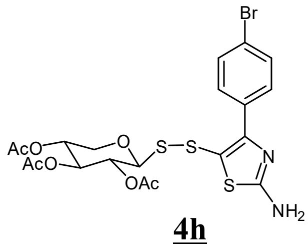

4h

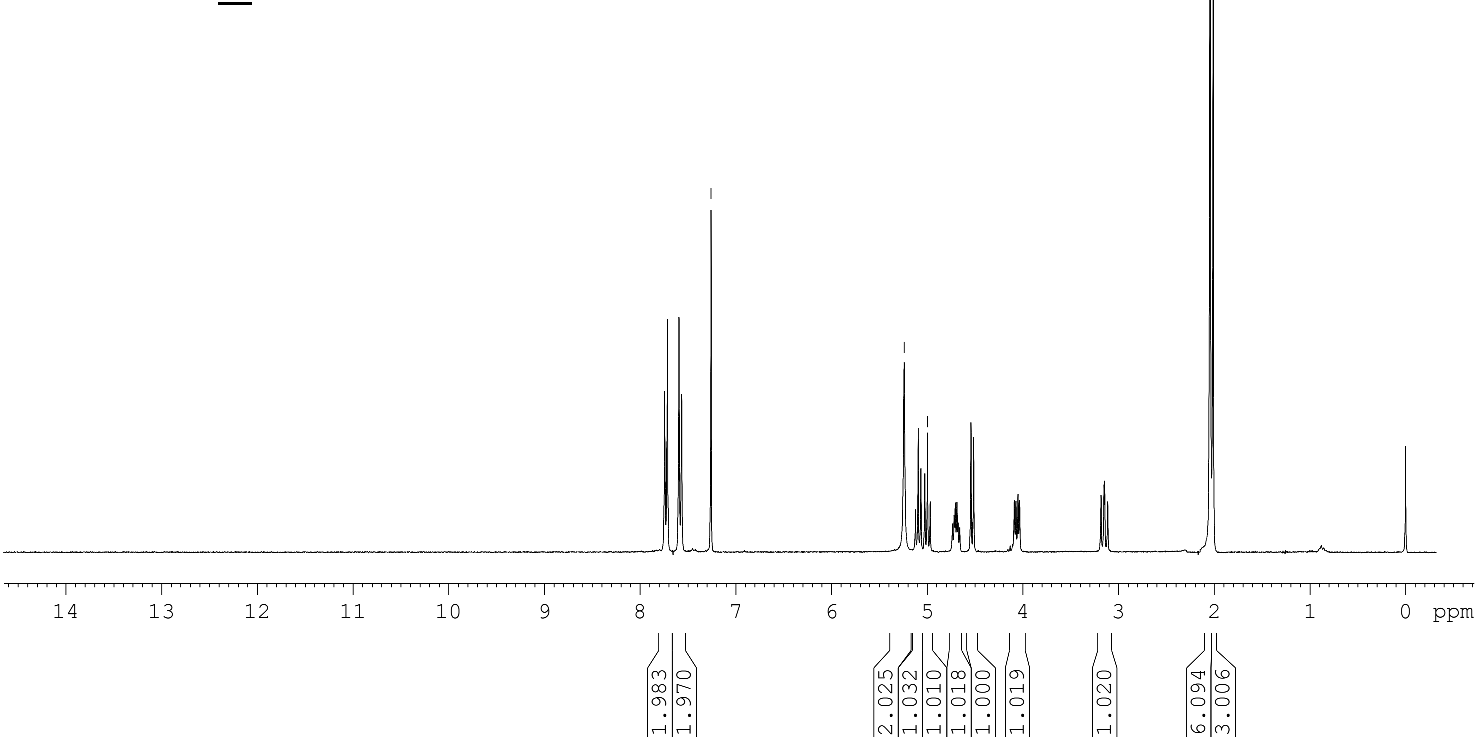

7.731
7.582
7.260
5.242
5.095
4.998
4.700
4.531
4.064
3.149
2.044
2.012
14 13 12 11 10 9 8 7 6 5 4 3 2 1 0 ppm
1.983
1.970
2.025
1.032
1.010
1.018
1.000
1.019
1.020
6.094
3.006

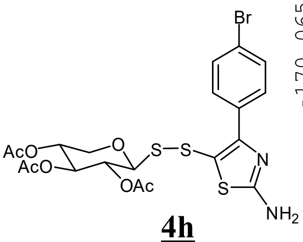

4h

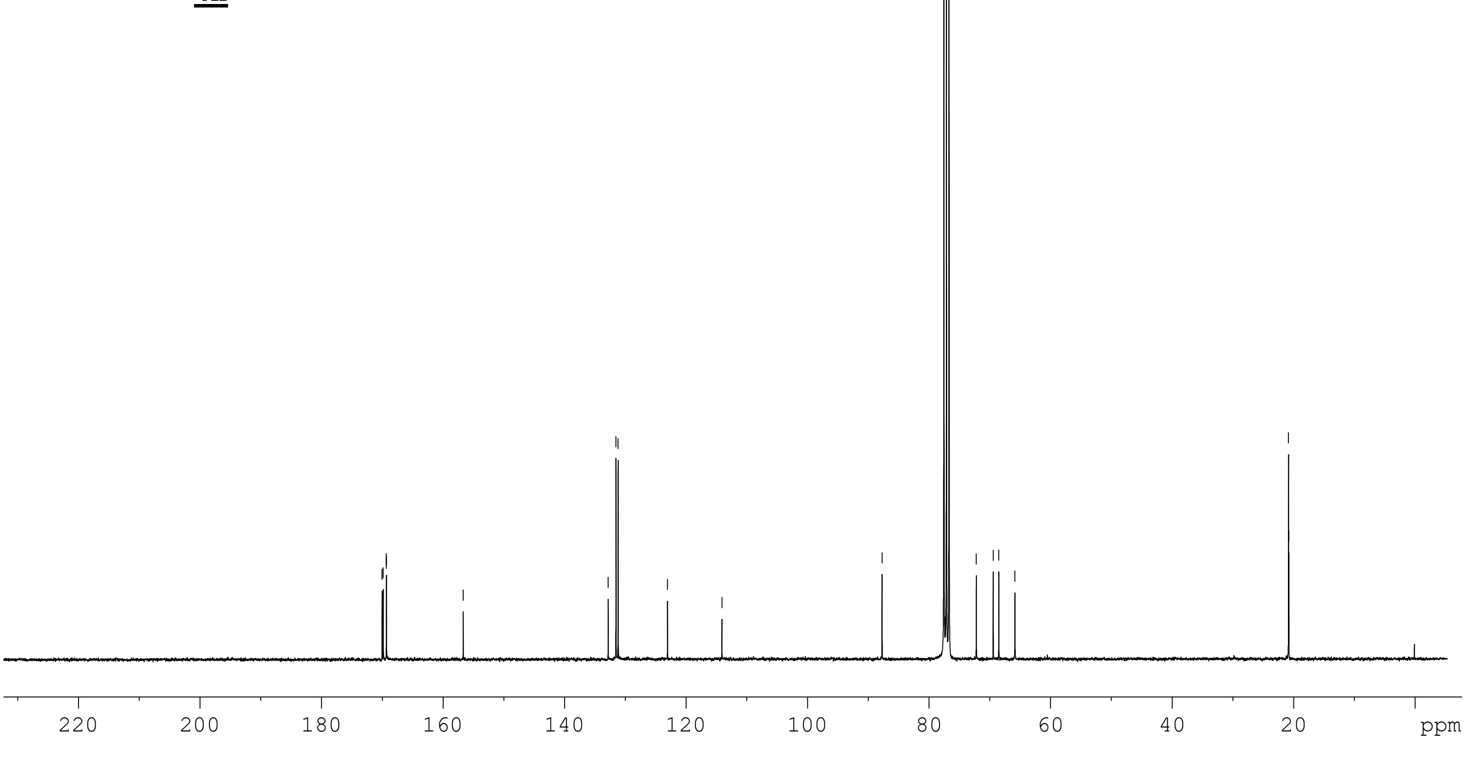

170.065
169.869
169.367
169.359
169.350
156.718
132.844
131.567
131.213
123.082
114.116
87.760
77.160
72.241
69.463
68.536
65.882
20.849
20.787
220 200 180 160 140 120 100 80 60 40 20 ppm

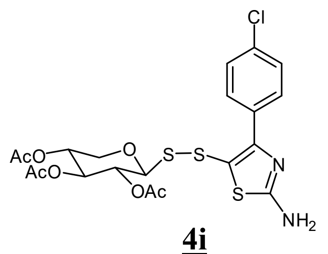
AcO
AcO
OAc
S–S
N
S
NH2
Cl

4i

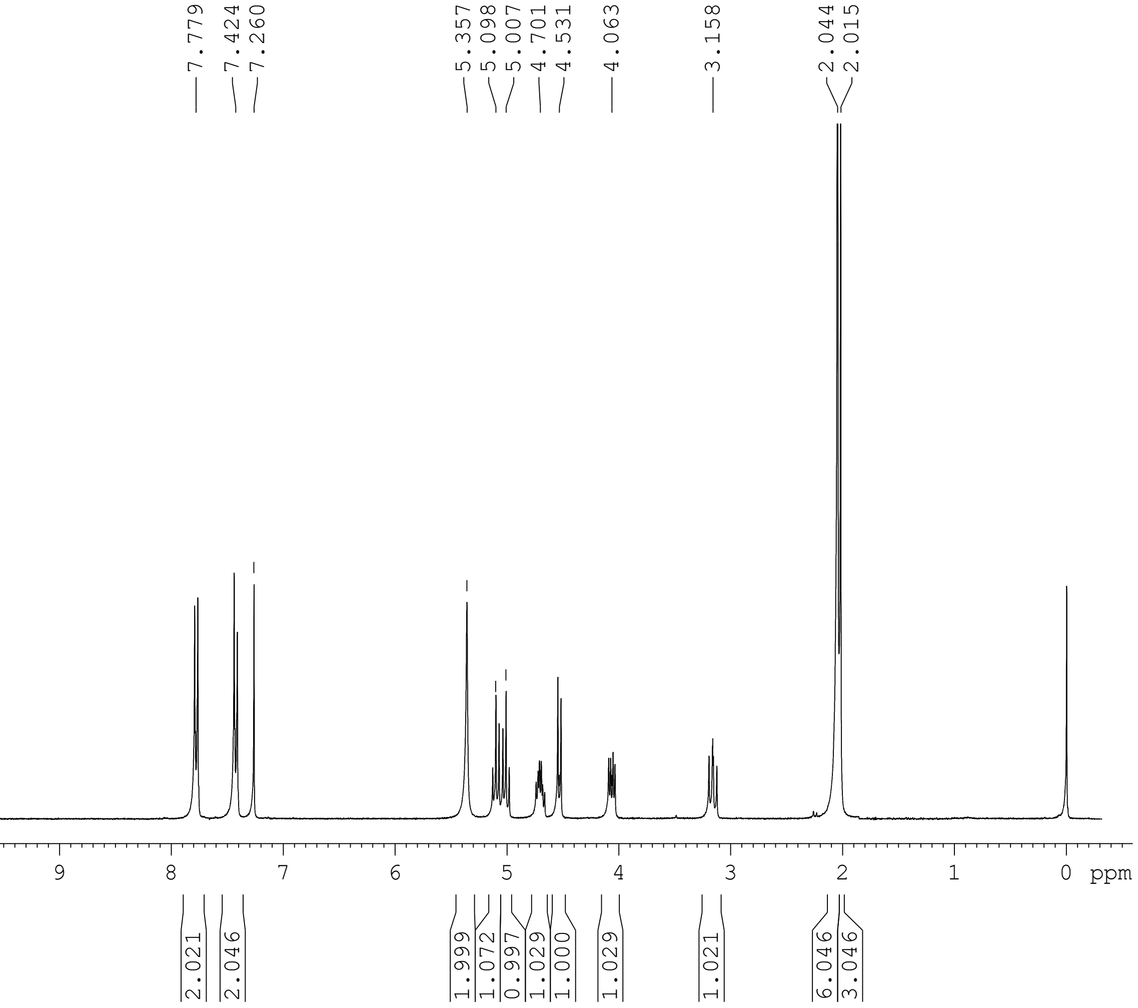
7.779
7.424
7.260
5.357
5.098
5.007
4.701
4.531
4.063
3.158
2.044
2.015
12 11 10 9 8 7 6 5 4 3 2 1 0 ppm
2.021
2.046
1.999
1.072
0.997
1.029
1.000
1.029
1.021
6.046
3.046

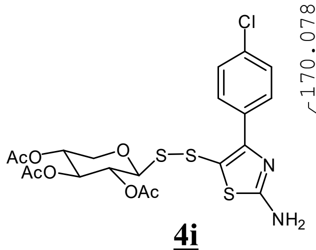
AcO
AcO
OAc
S–S
N
S
NH2
Cl

4i

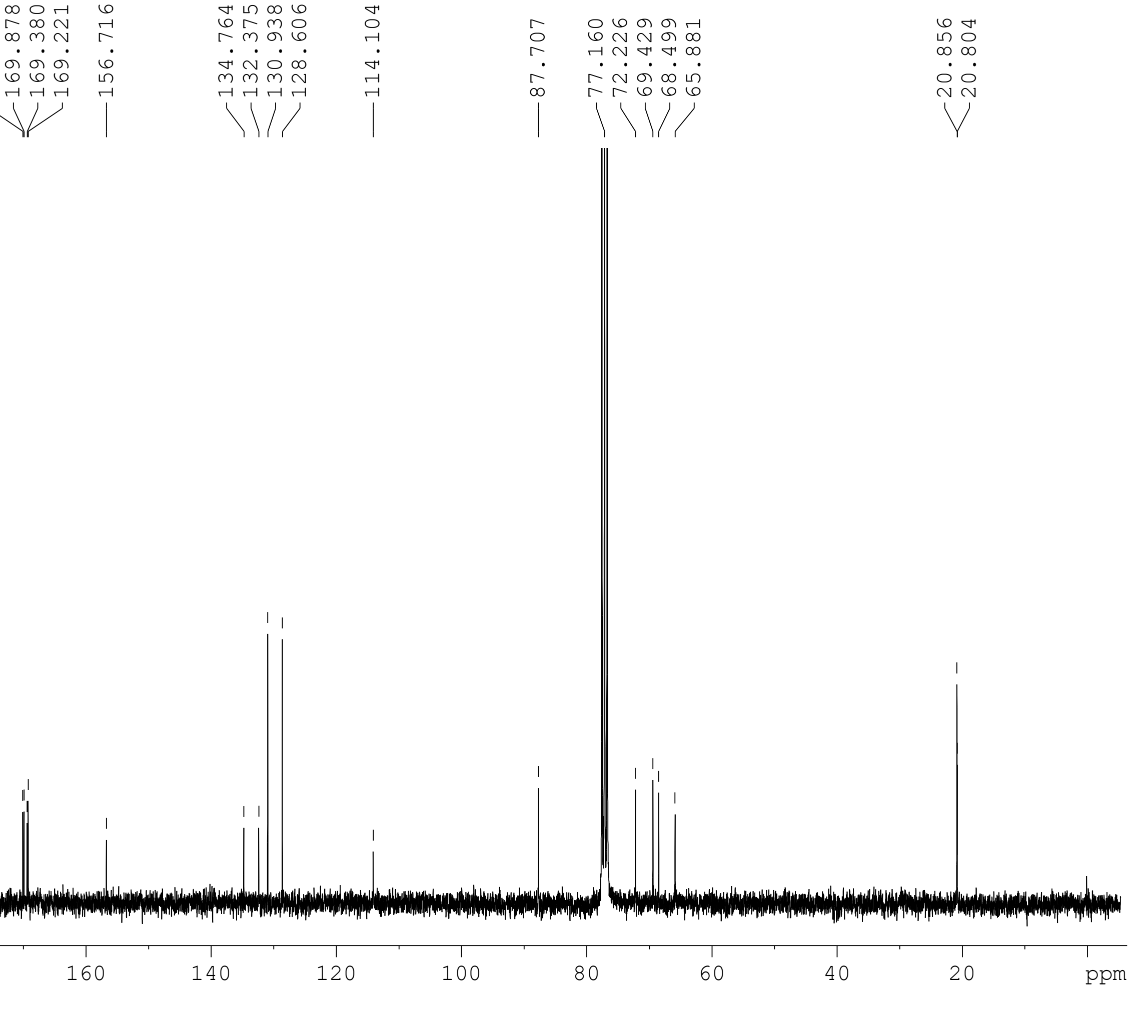
170.078
169.878
169.380
169.221
156.716
134.764
132.375
130.938
128.606
114.104
87.707
77.160
72.226
69.429
68.499
65.881
20.856
20.804
220 200 180 160 140 120 100 80 60 40 20 ppm

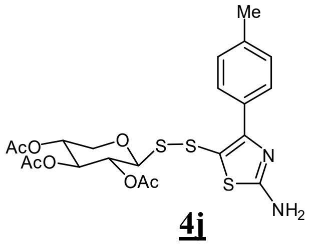

4i

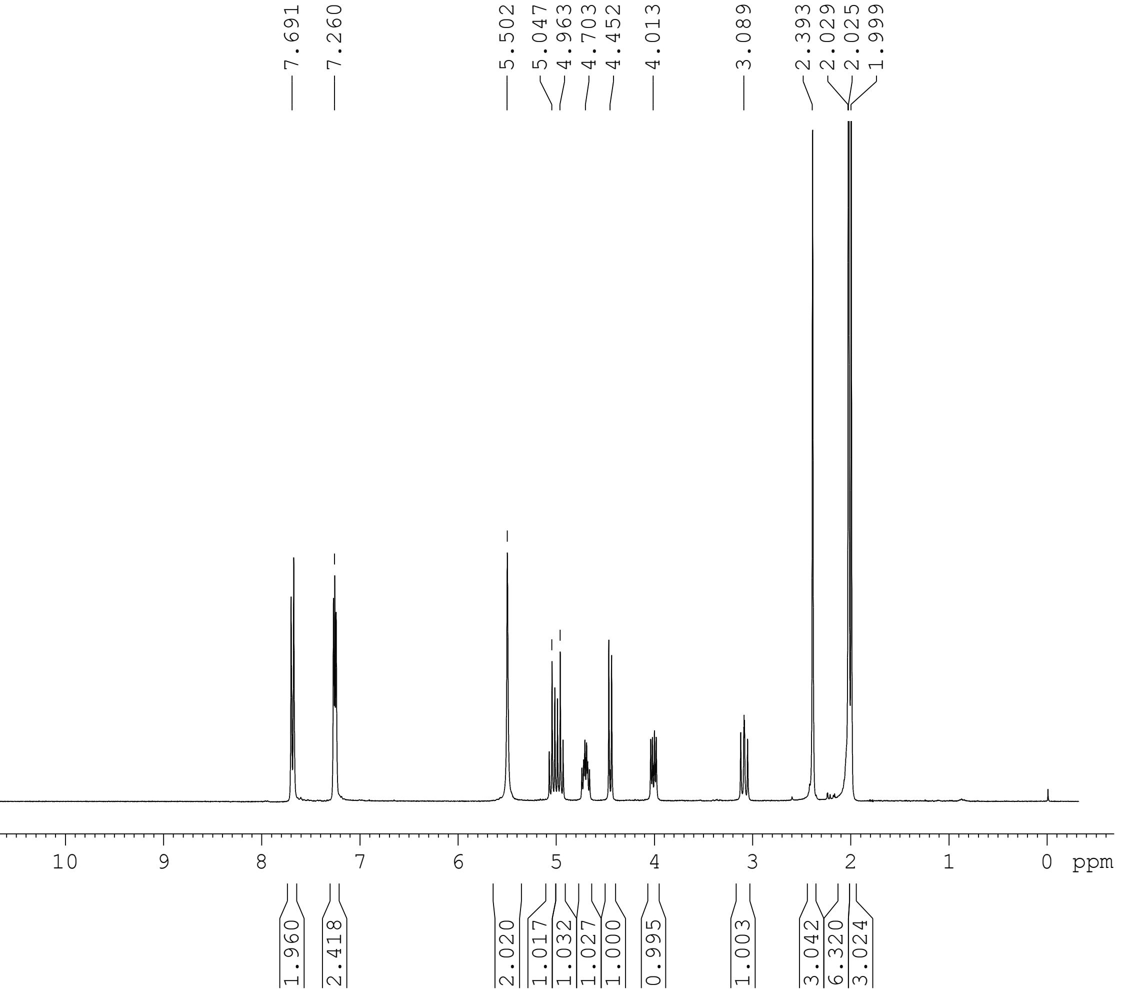
7.691
7.260
5.502
5.047
4.963
4.703
4.452
4.013
3.089
2.393
2.029
2.025
1.999
14 13 12 11 10 9 8 7 6 5 4 3 2 1 0 ppm
1.960
2.418
2.020
1.017
1.032
1.027
1.000
0.995
1.003
3.042
6.320
3.024

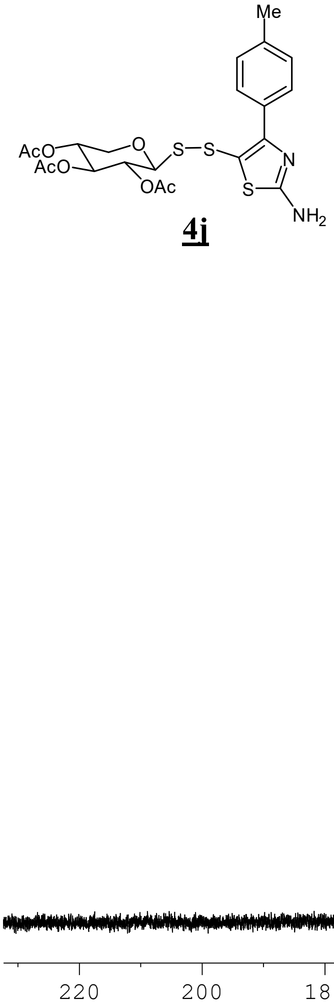

4i

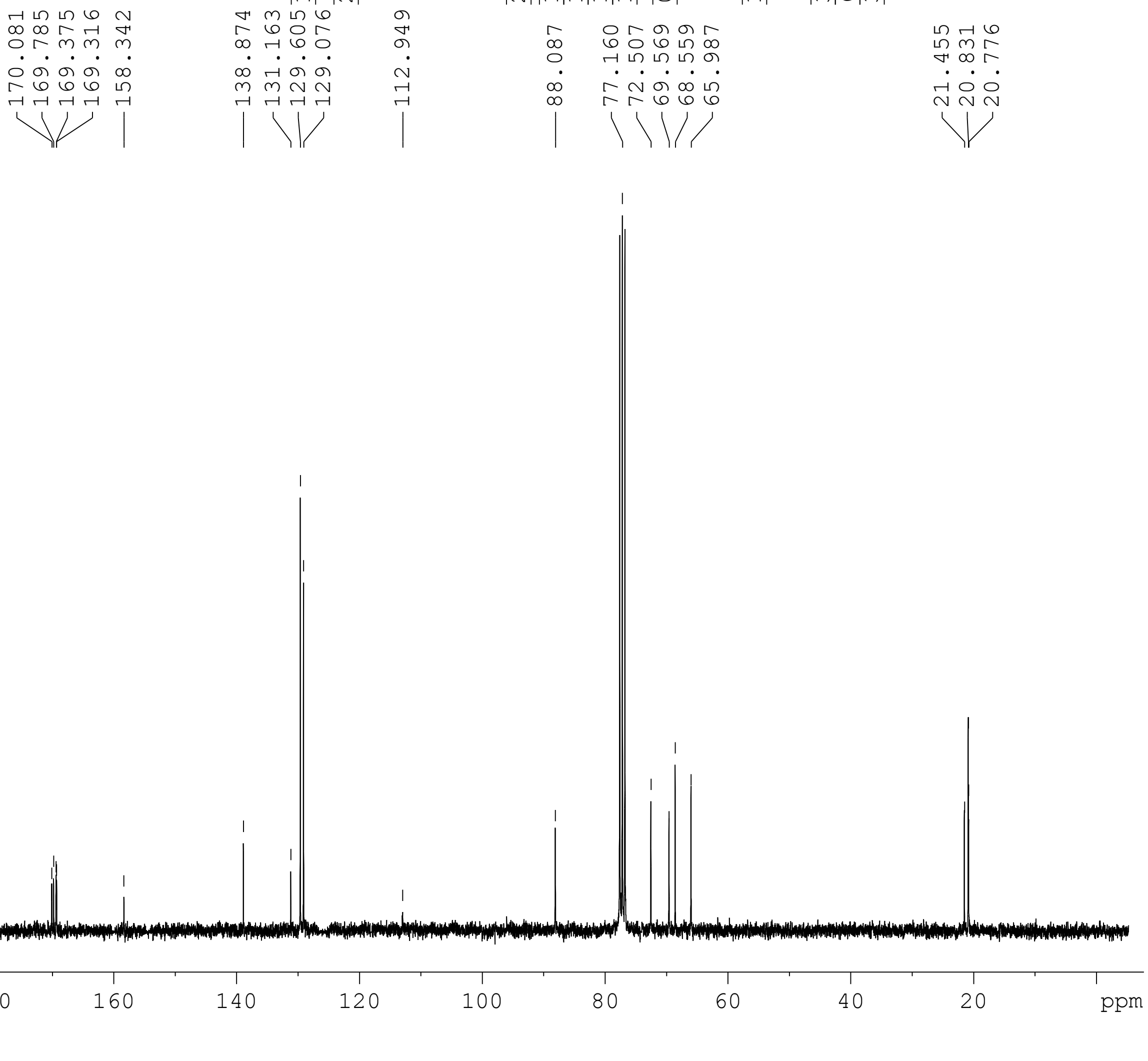
170.081
169.785
169.375
169.316
158.342
138.874
131.163
129.605
129.076
112.949
88.087
77.160
72.507
69.569
68.559
65.987
21.455
20.831
20.776
220 200 180 160 140 120 100 80 60 40 20 ppm

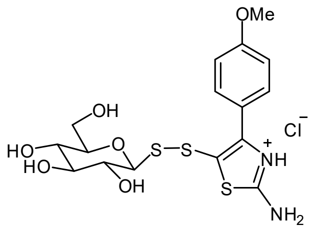
OMe
OH
HO
HO
O
OH
S
S
S
NH
+
Cl−
NH2

5a

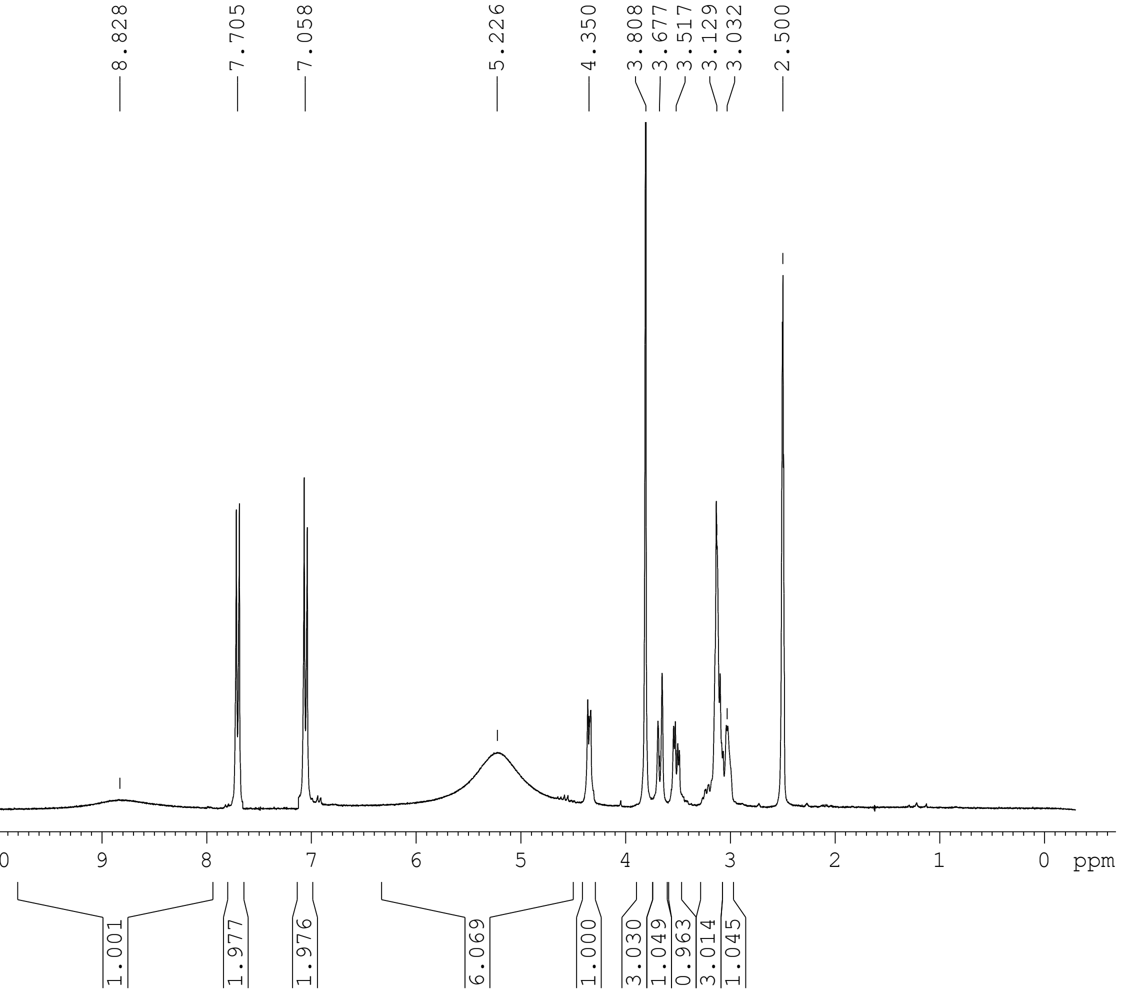
8.828
7.705
7.058
5.226
4.350
3.808
3.677
3.517
3.129
3.032
2.500
13 12 11 10 9 8 7 6 5 4 3 2 1 0 ppm
1.001
1.977
1.976
6.069
1.000
3.030
1.049
0.963
3.014
1.045

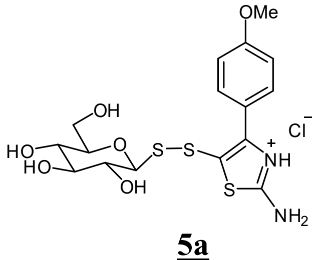
OMe
OH
HO
HO
O
OH
S
S
S
NH
+
Cl−
NH2

5a

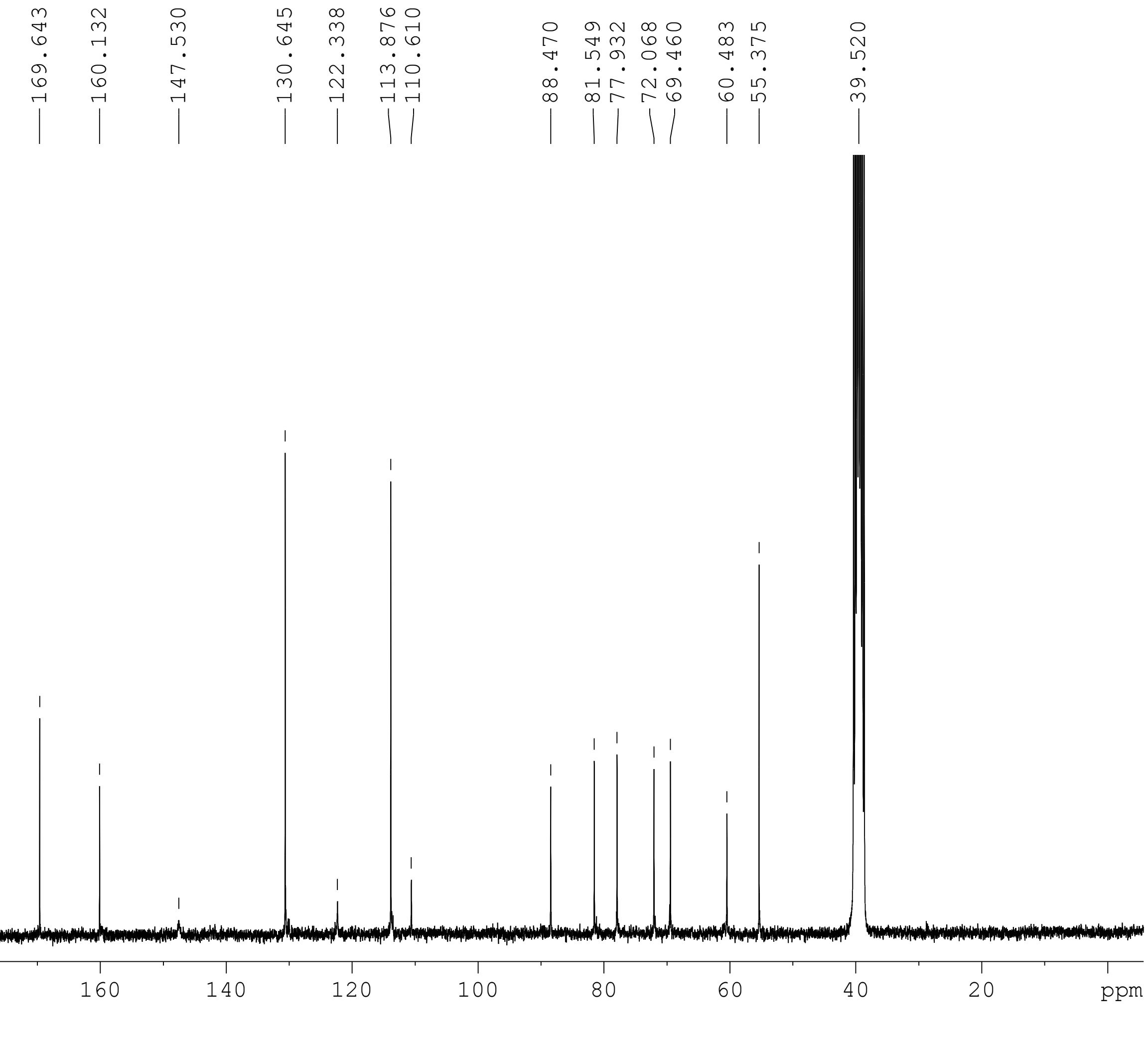
169.643
160.132
147.530
130.645
122.338
113.876
110.610
88.470
81.549
77.932
72.068
69.460
60.483
55.375
39.520
220 200 180 160 140 120 100 80 60 40 20 ppm

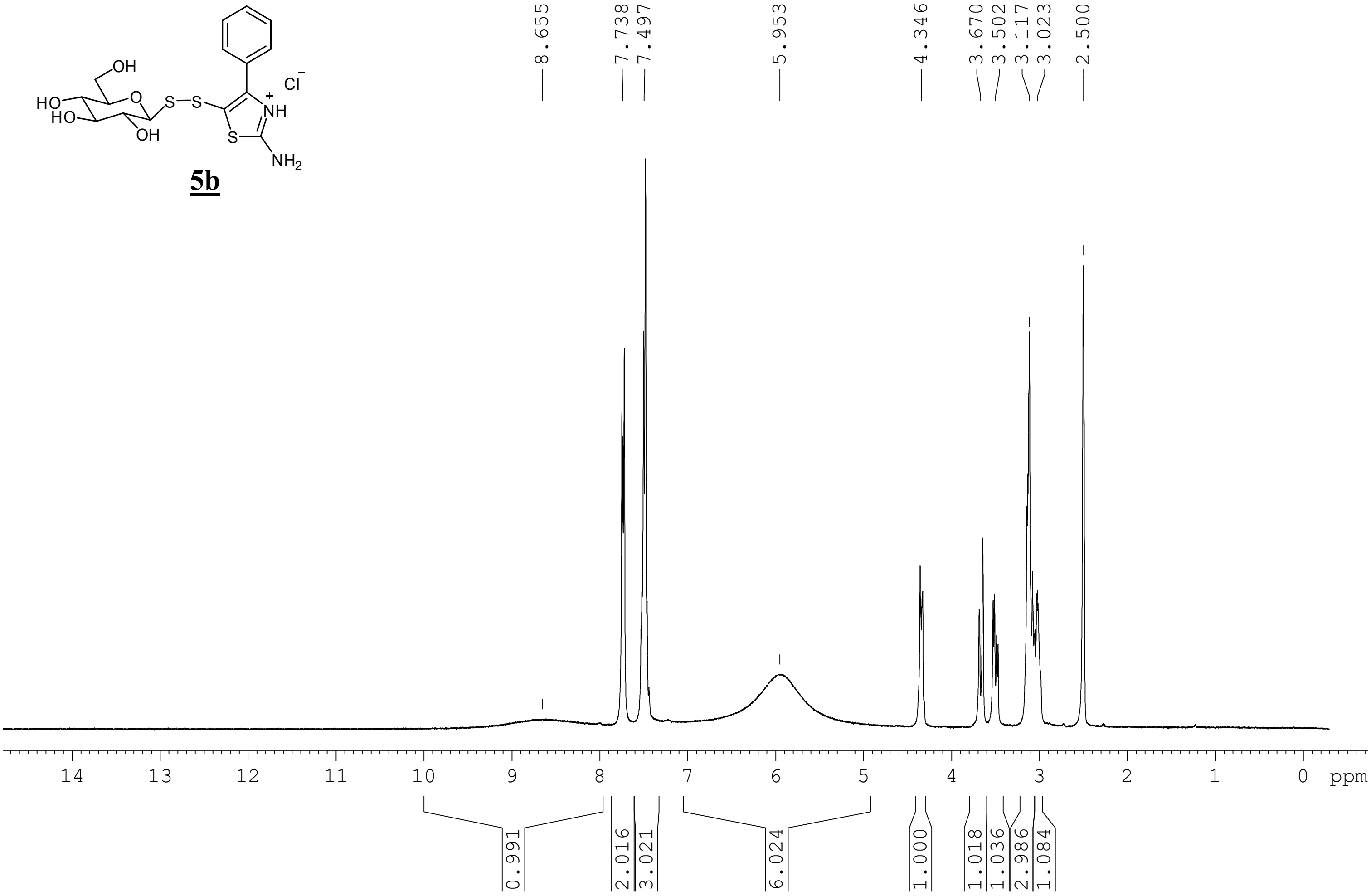

OH
HO
HO
O
OH
S–S
S
NH
NH2
Cl−
5b
8.655
7.738
7.497
5.953
4.346
3.670
3.502
3.117
3.023
2.500
14 13 12 11 10 9 8 7 6 5 4 3 2 1 0 ppm
0.991
2.016
3.021
6.024
1.000
1.018
1.036
2.986
1.084

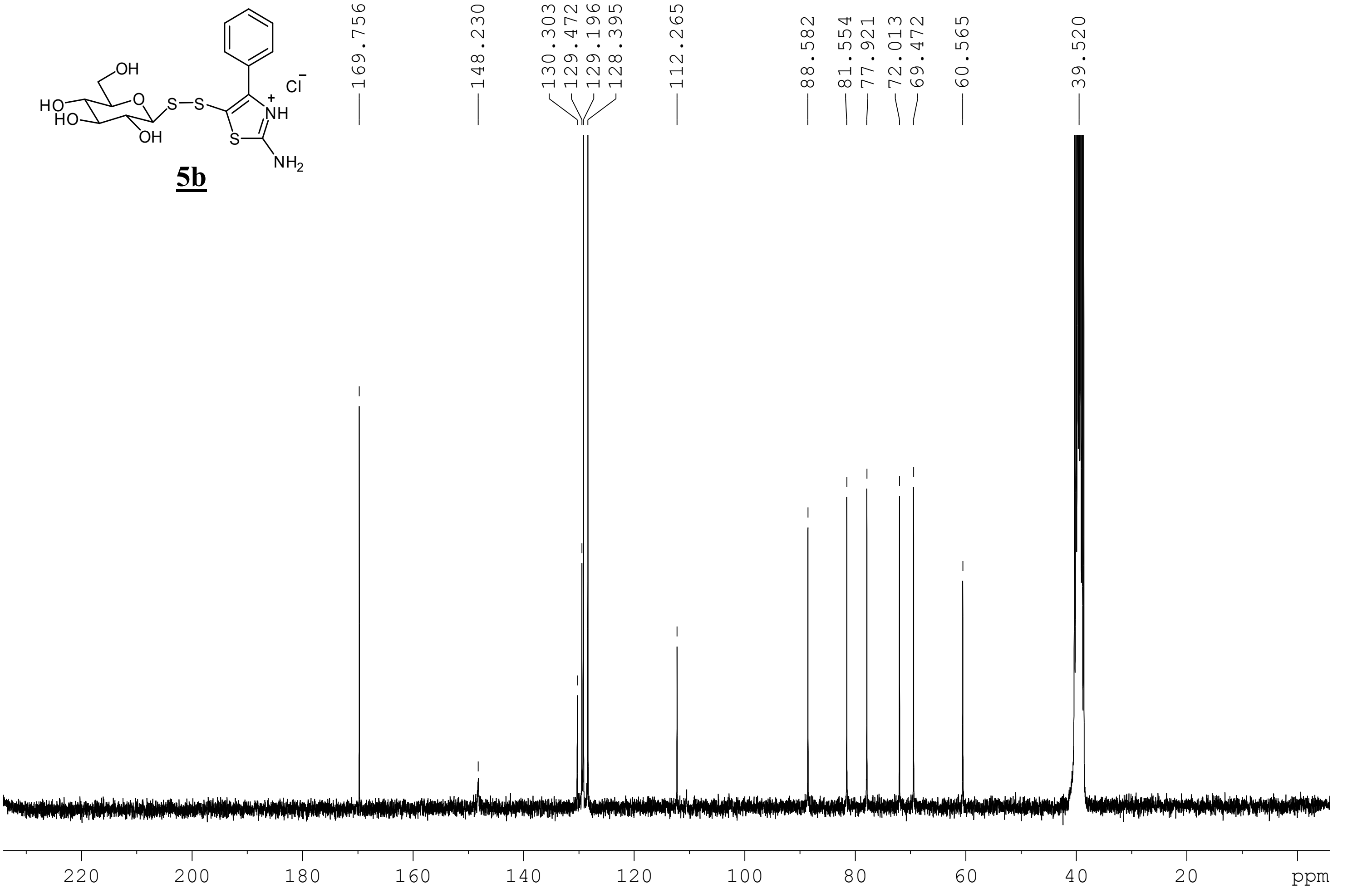

OH
HO
HO
O
OH
S–S
S
NH
NH2
Cl−
5b
169.756
148.230
130.303
129.472
129.196
128.395
112.265
88.582
81.554
77.921
72.013
69.472
60.565
39.520
220 200 180 160 140 120 100 80 60 40 20 ppm

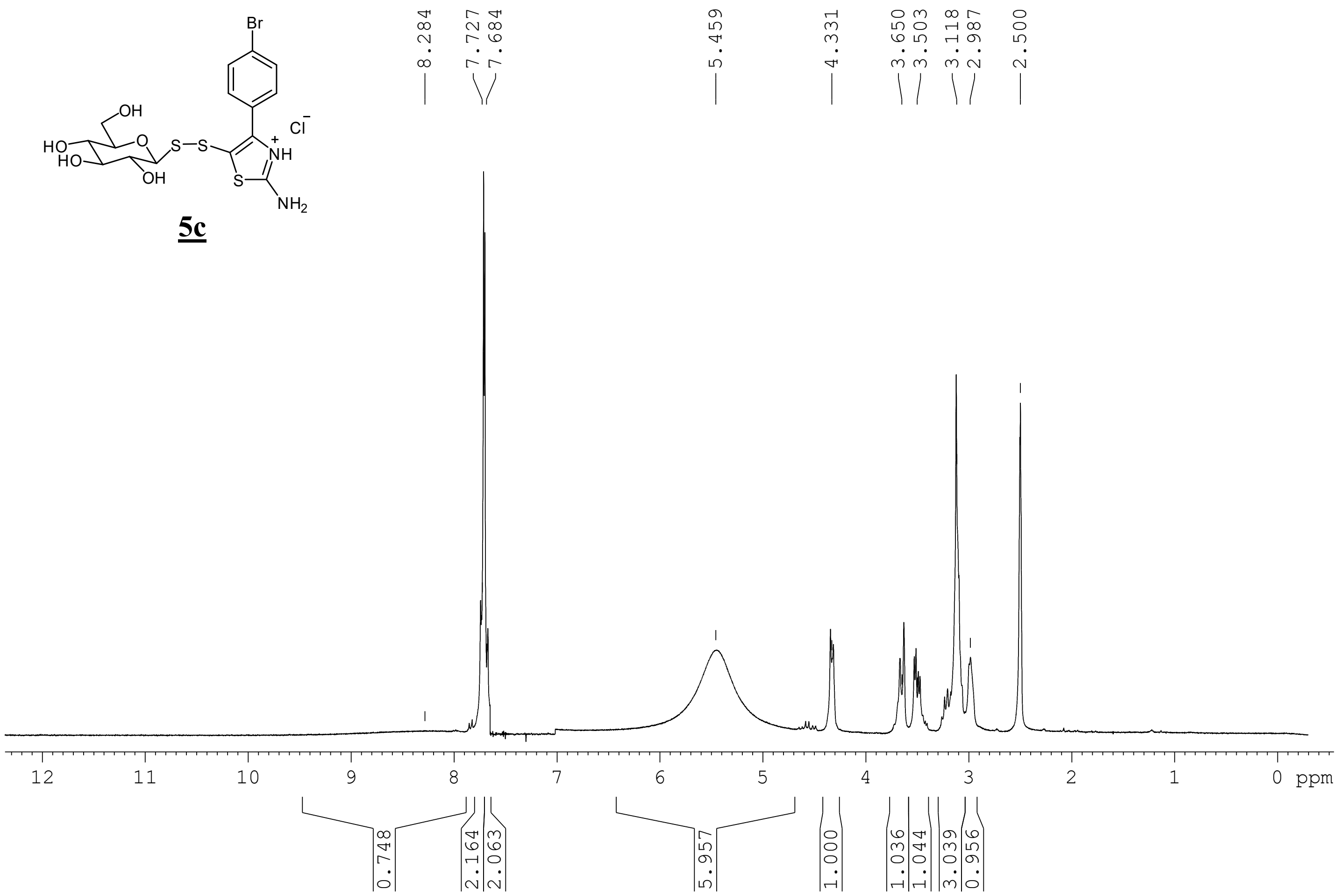
Br
OH
HO
HO
O
S–S
OH
Cl⁻
NH
S
NH2
5c
8.284
7.727
7.684
5.459
4.331
3.650
3.503
3.118
2.987
2.500
12 11 10 9 8 7 6 5 4 3 2 1 0 ppm
0.748
2.164
2.063
5.957
1.000
1.036
1.044
3.039
0.956

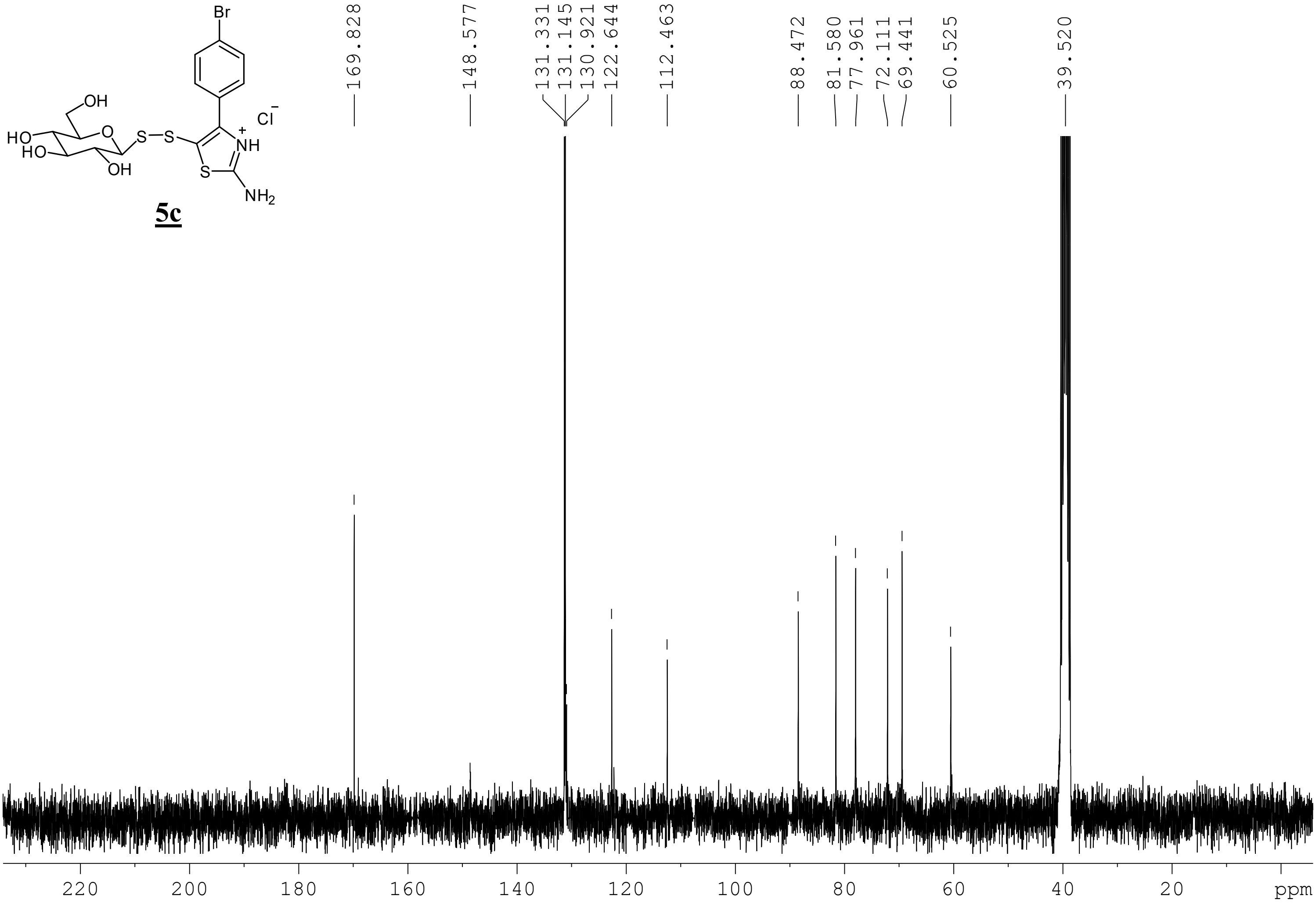
Br
OH
HO
HO
O
S–S
OH
Cl⁻
NH
S
NH2
5c
169.828
148.577
131.331
131.145
130.921
122.644
112.463
88.472
81.580
77.961
72.111
69.441
60.525
39.520
220 200 180 160 140 120 100 80 60 40 20 ppm

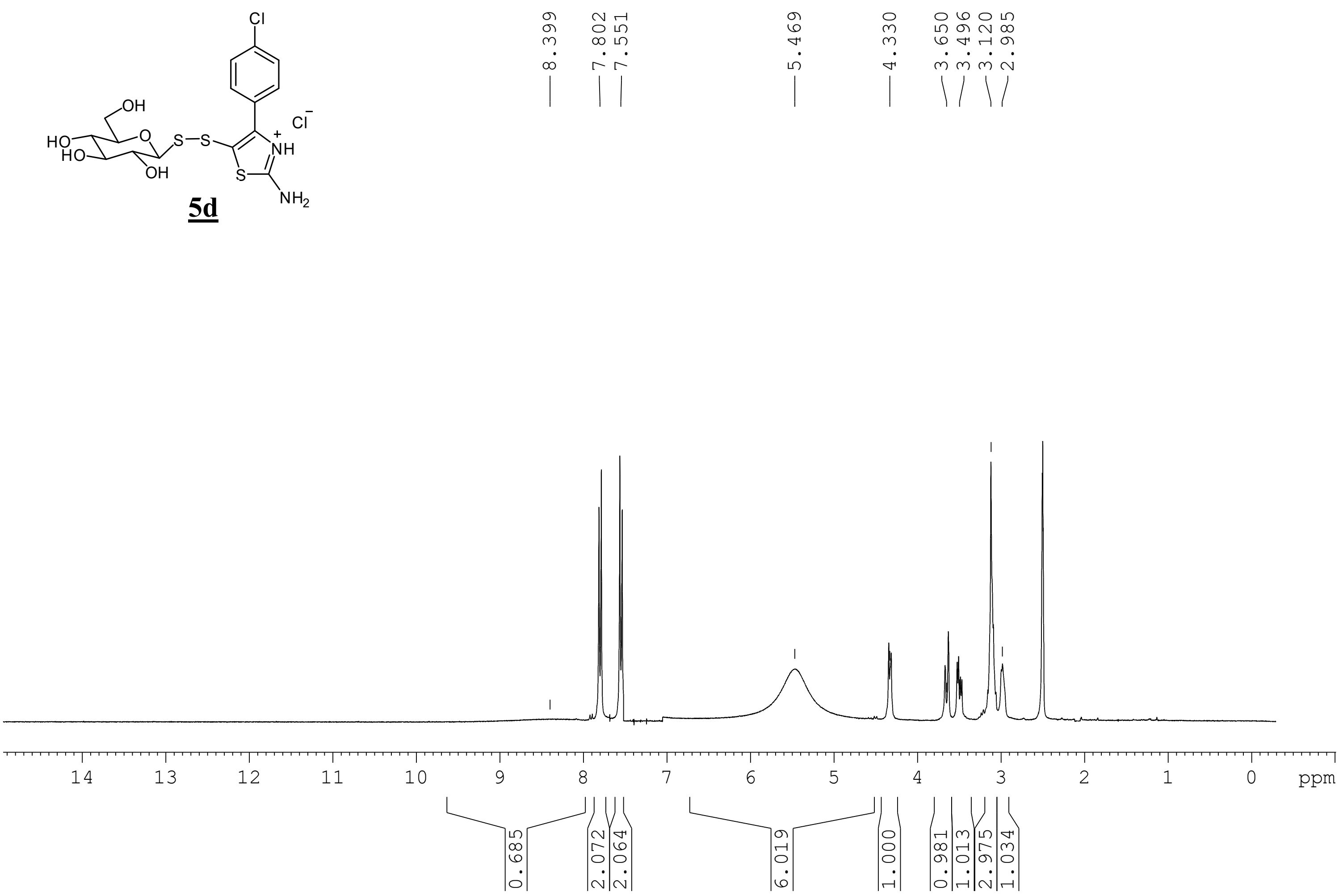
5d
8.399
7.802
7.551
5.469
4.330
3.650
3.496
3.120
2.985
14 13 12 11 10 9 8 7 6 5 4 3 2 1 0 ppm
0.685
2.072
2.064
6.019
1.000
0.981
1.013
2.975
1.034

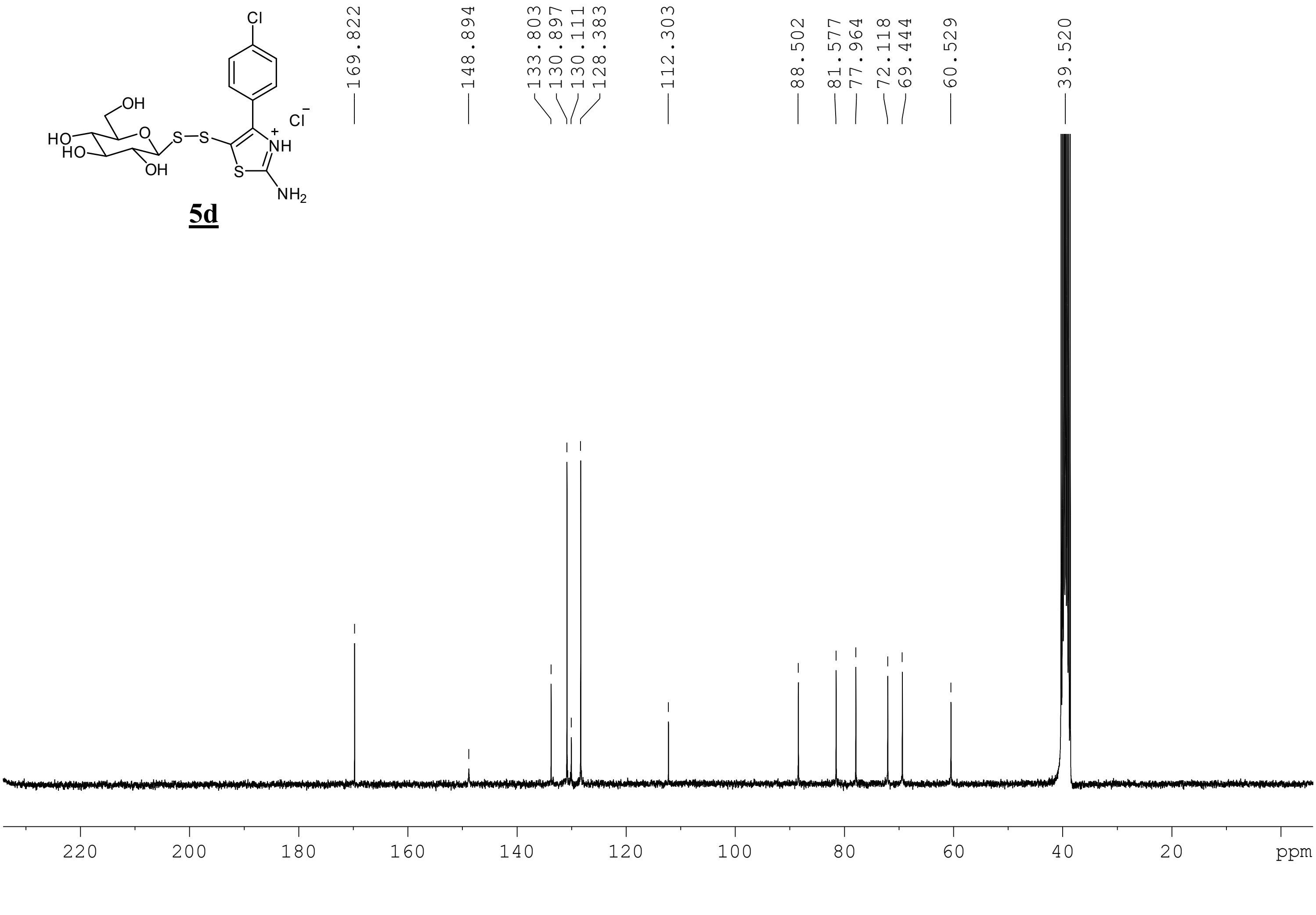
5d
169.822
148.894
133.803
130.897
130.111
128.383
112.303
88.502
81.577
77.964
72.118
69.444
60.529
39.520
220 200 180 160 140 120 100 80 60 40 20 ppm

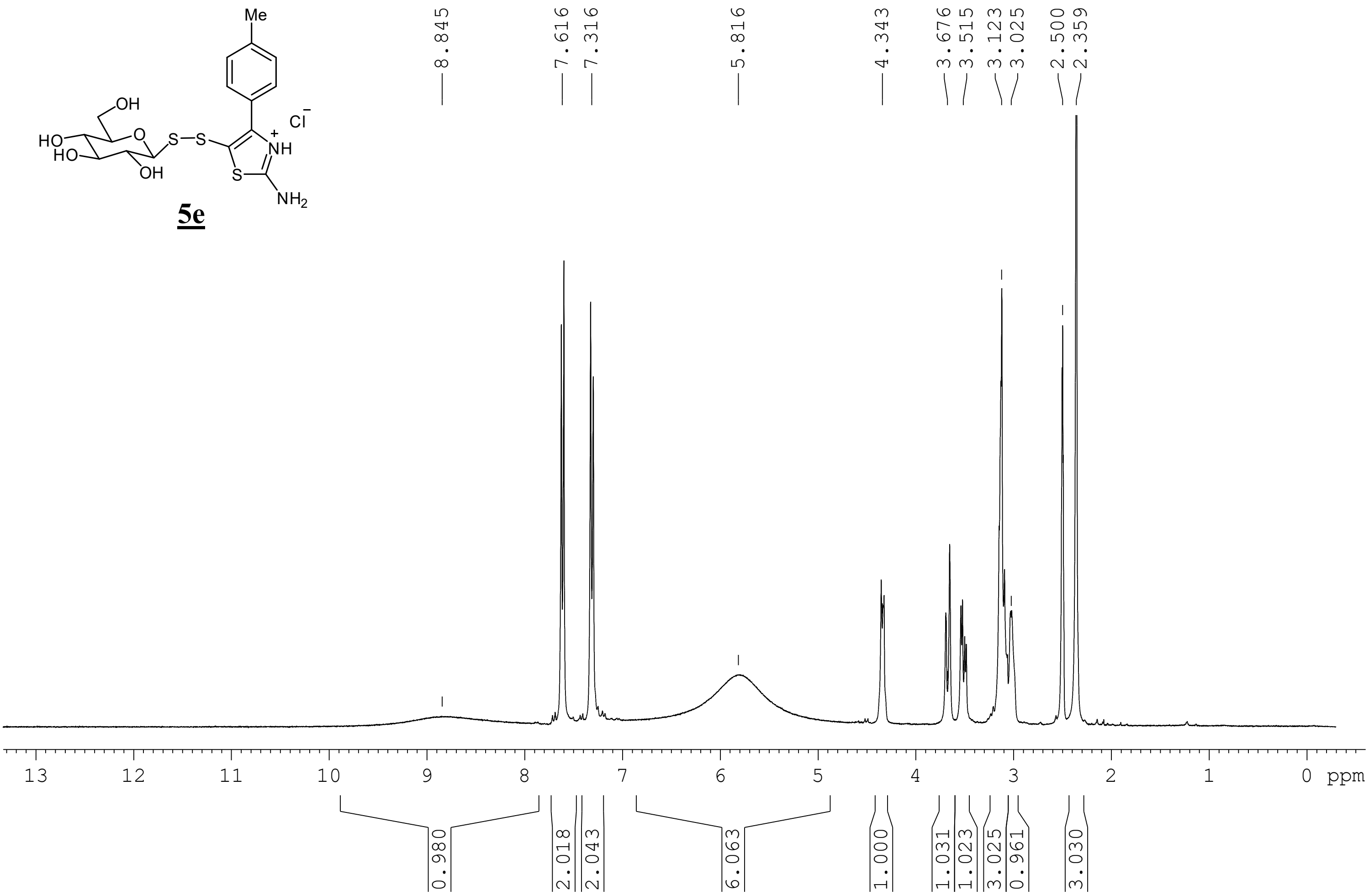

Me
OH
HO
HO
O
S–S
OH
Cl−
NH
S
NH2
5e
8.845
7.616
7.316
5.816
4.343
3.676
3.515
3.123
3.025
2.500
2.359
13 12 11 10 9 8 7 6 5 4 3 2 1 0 ppm
0.980
2.018
2.043
6.063
1.000
1.031
1.023
3.025
0.961
3.030

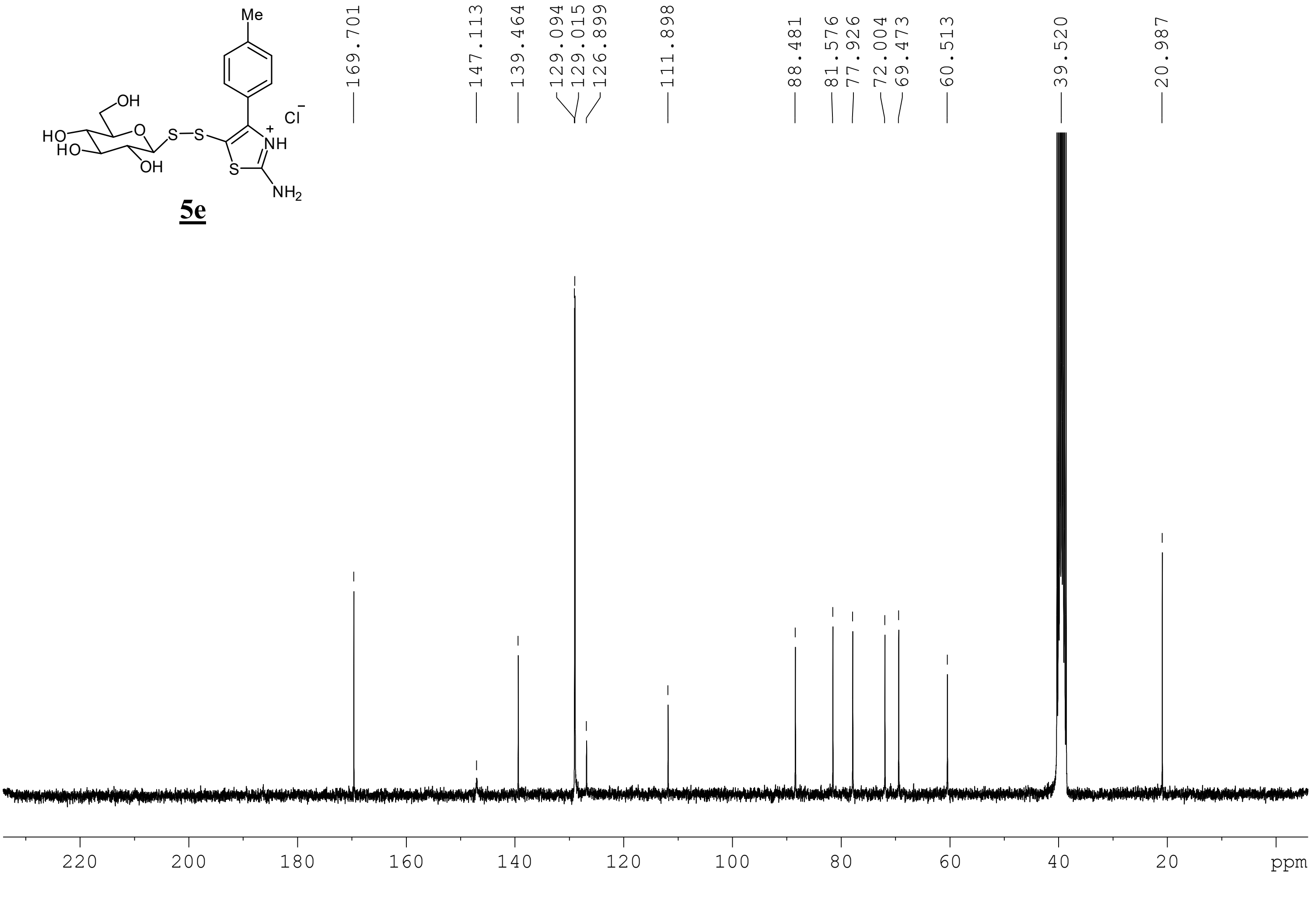

Me
OH
HO
HO
O
S–S
OH
Cl−
NH
S
NH2
5e
169.701
147.113
139.464
129.094
129.015
126.899
111.898
88.481
81.576
77.926
72.004
69.473
60.513
39.520
20.987
220 200 180 160 140 120 100 80 60 40 20 ppm

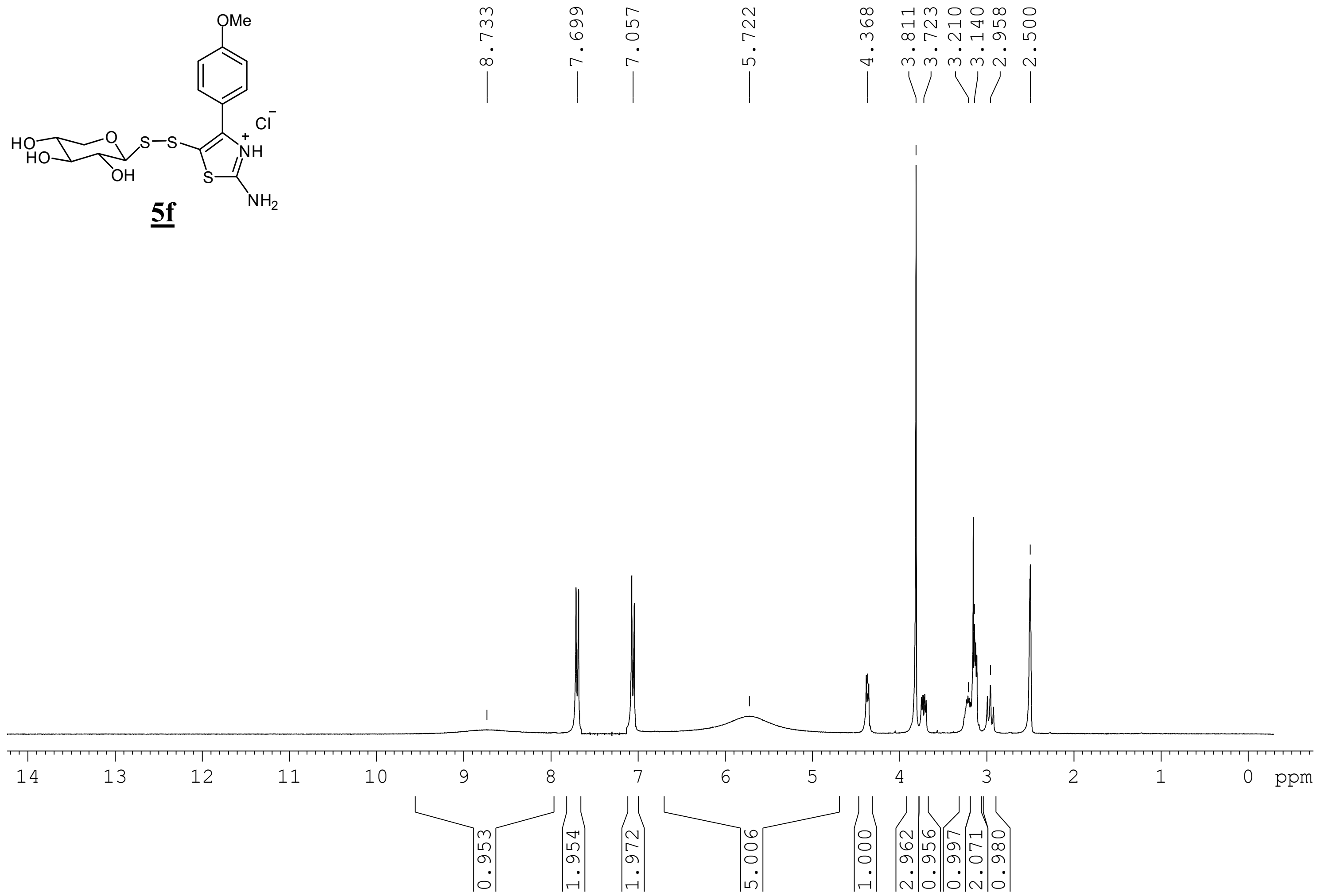
OMe
HO
HO
O
OH
S—S
S
NH
Cl
NH2
5f
8.733
7.699
7.057
5.722
4.368
3.811
3.723
3.210
3.140
2.958
2.500
14 13 12 11 10 9 8 7 6 5 4 3 2 1 0 ppm
0.953
1.954
1.972
5.006
1.000
2.962
0.956
0.997
2.071
0.980

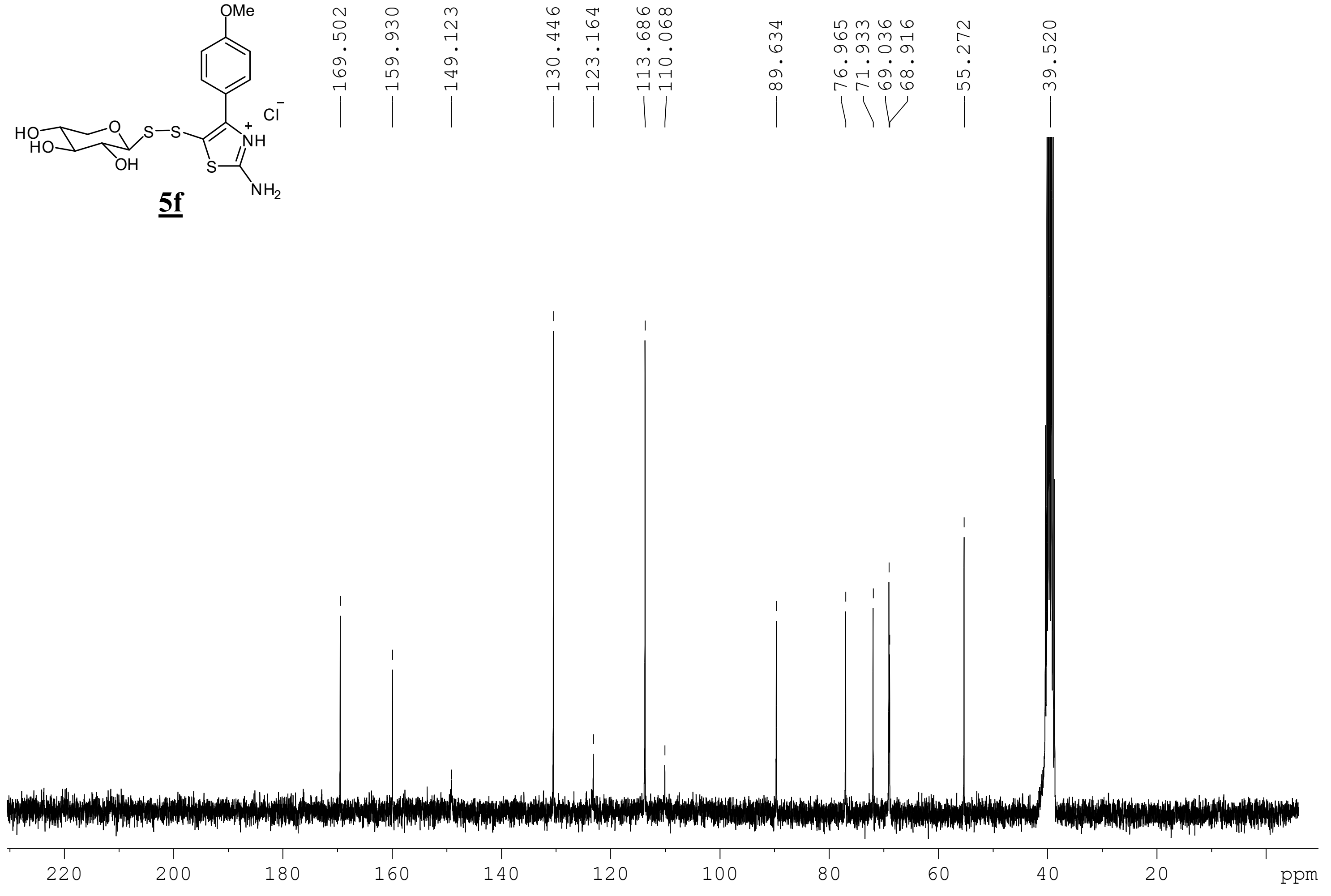
OMe
HO
HO
O
OH
S—S
S
NH
Cl
NH2
5f
169.502
159.930
149.123
130.446
123.164
113.686
110.068
89.634
76.965
71.933
69.036
68.916
55.272
39.520
220 200 180 160 140 120 100 80 60 40 20 ppm

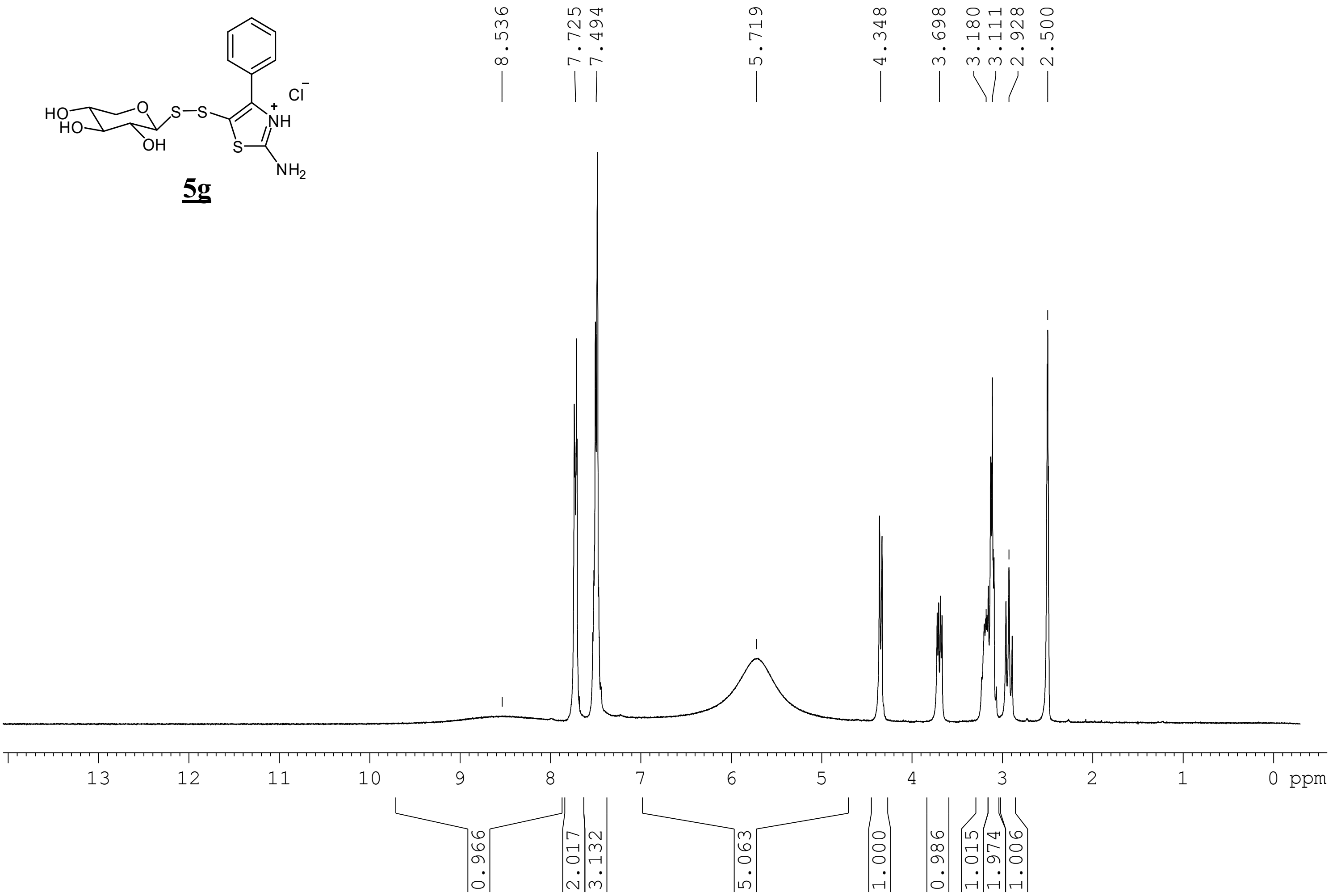

HO
HO
OH
O
S
S
S
NH
NH2
Cl
8.536
7.725
7.494
5.719
4.348
3.698
3.180
3.111
2.928
2.500
0.966
2.017
3.132
5.063
1.000
0.986
1.015
1.974
1.006
13
12
11
10
9
8
7
6
5
4
3
2
1
0 ppm

5g

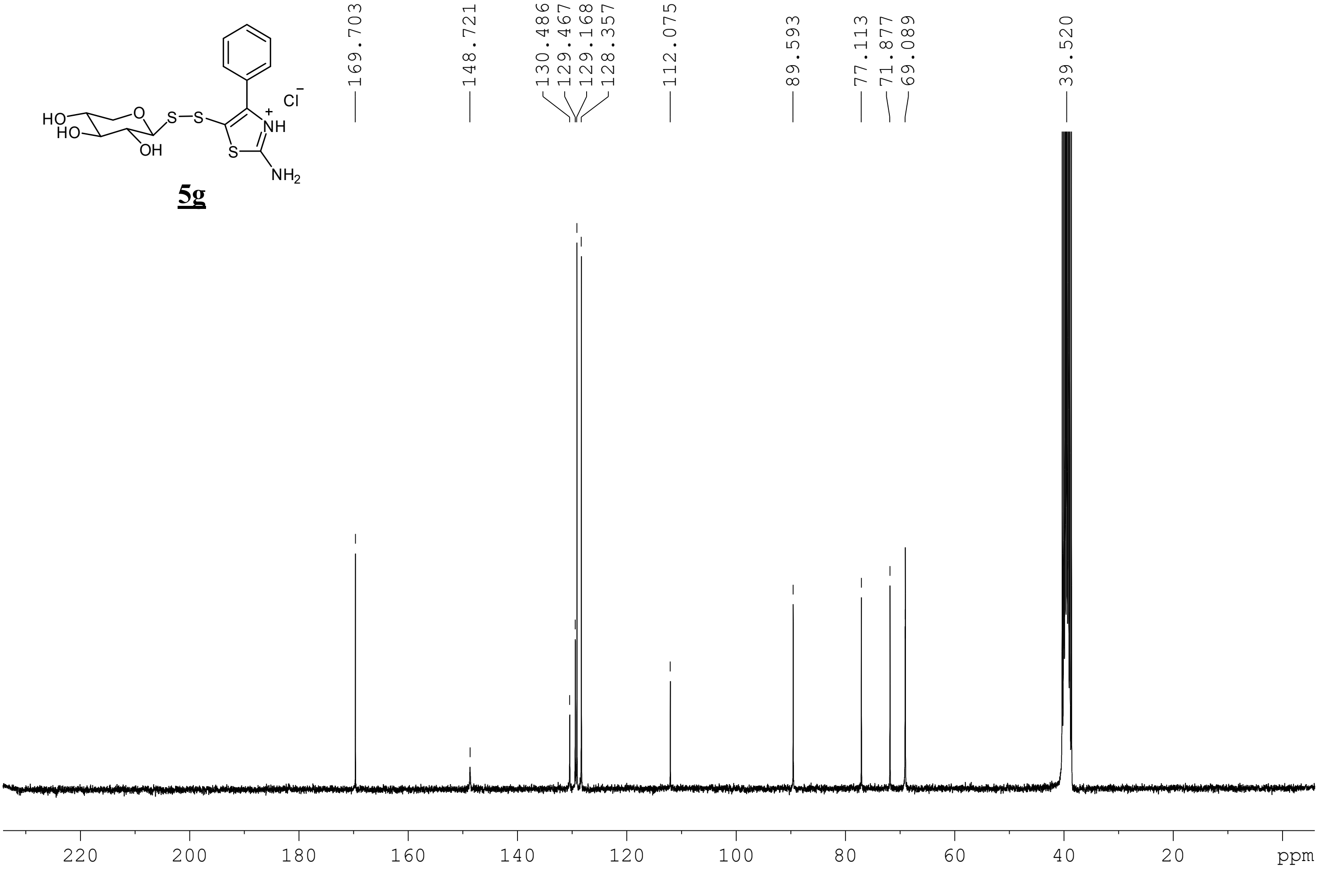

HO
HO
OH
O
S
S
S
NH
NH2
Cl
169.703
148.721
130.486
129.467
129.168
128.357
112.075
89.593
77.113
71.877
69.089
39.520
220
200
180
160
140
120
100
80
60
40
20
ppm

5g

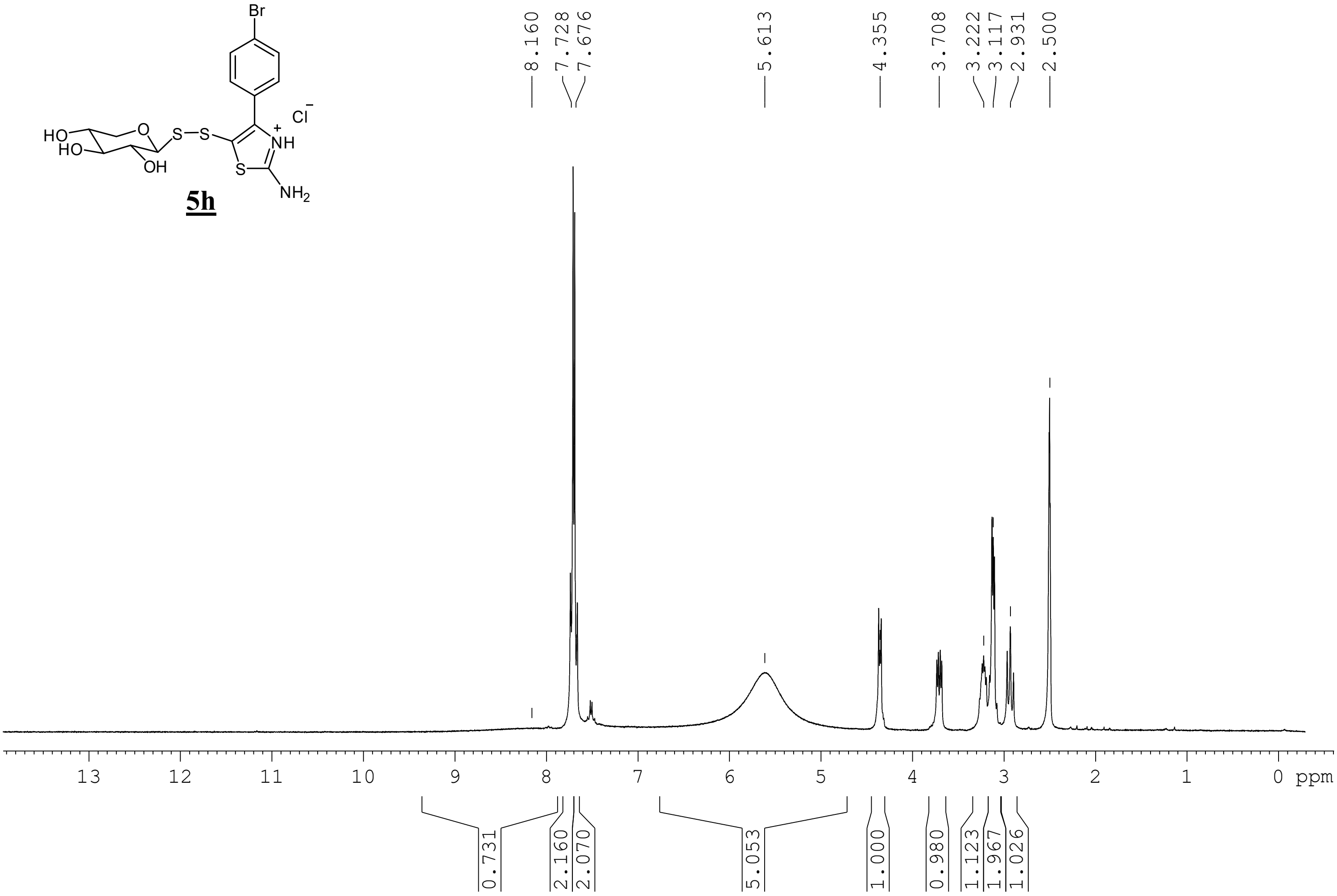
Br
Cl⁻
HO
HO
OH
S–S
NH
S
NH2
5h
8.160
7.728
7.676
5.613
4.355
3.708
3.222
3.117
2.931
2.500
13 12 11 10 9 8 7 6 5 4 3 2 1 0 ppm
0.731
2.160
2.070
5.053
1.000
0.980
1.123
1.967
1.026

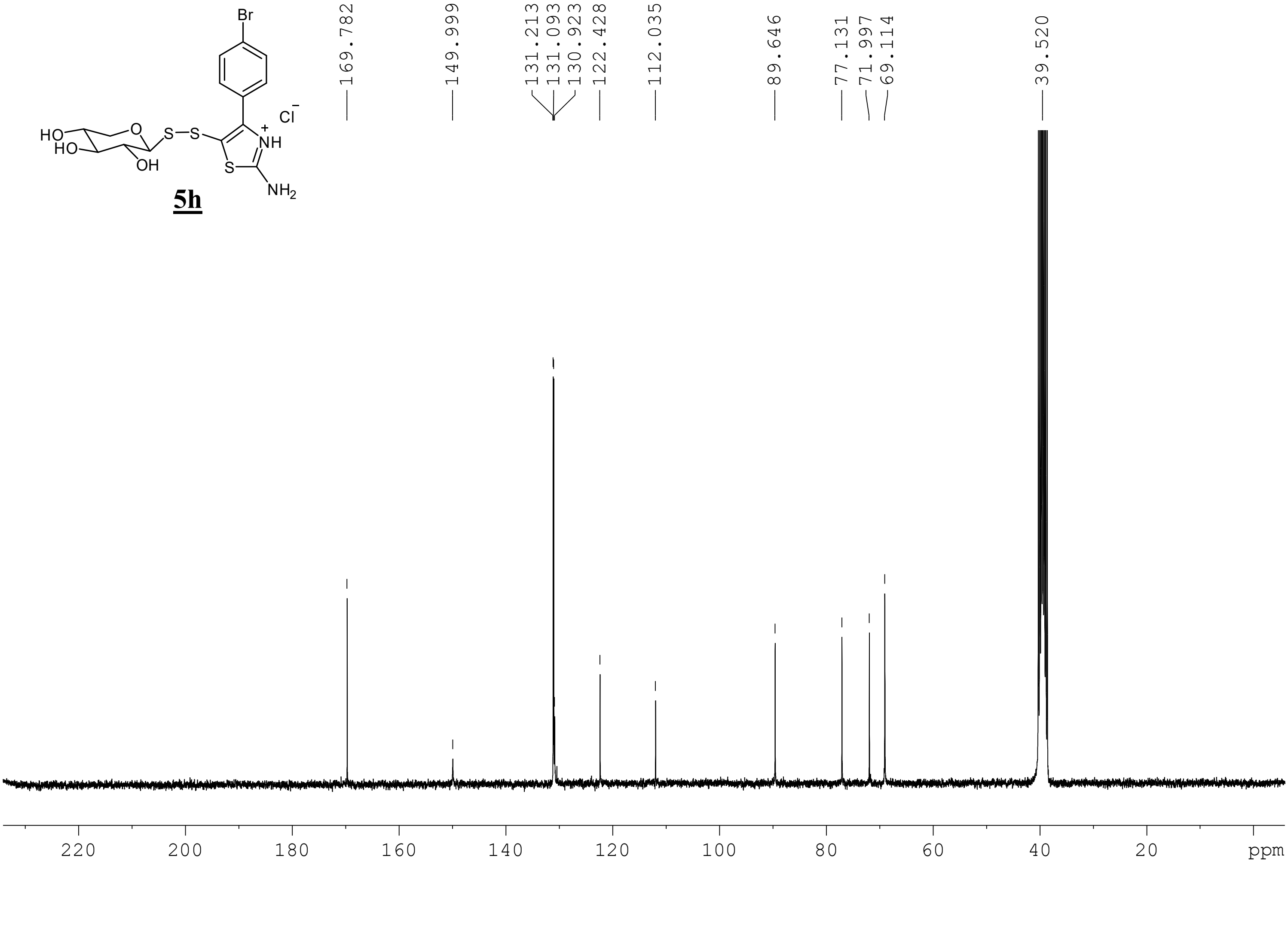
Br
Cl⁻
HO
HO
OH
S–S
NH
S
NH2
5h
169.782
149.999
131.213
131.093
130.923
122.428
112.035
89.646
77.131
71.997
69.114
39.520
220 200 180 160 140 120 100 80 60 40 20 ppm

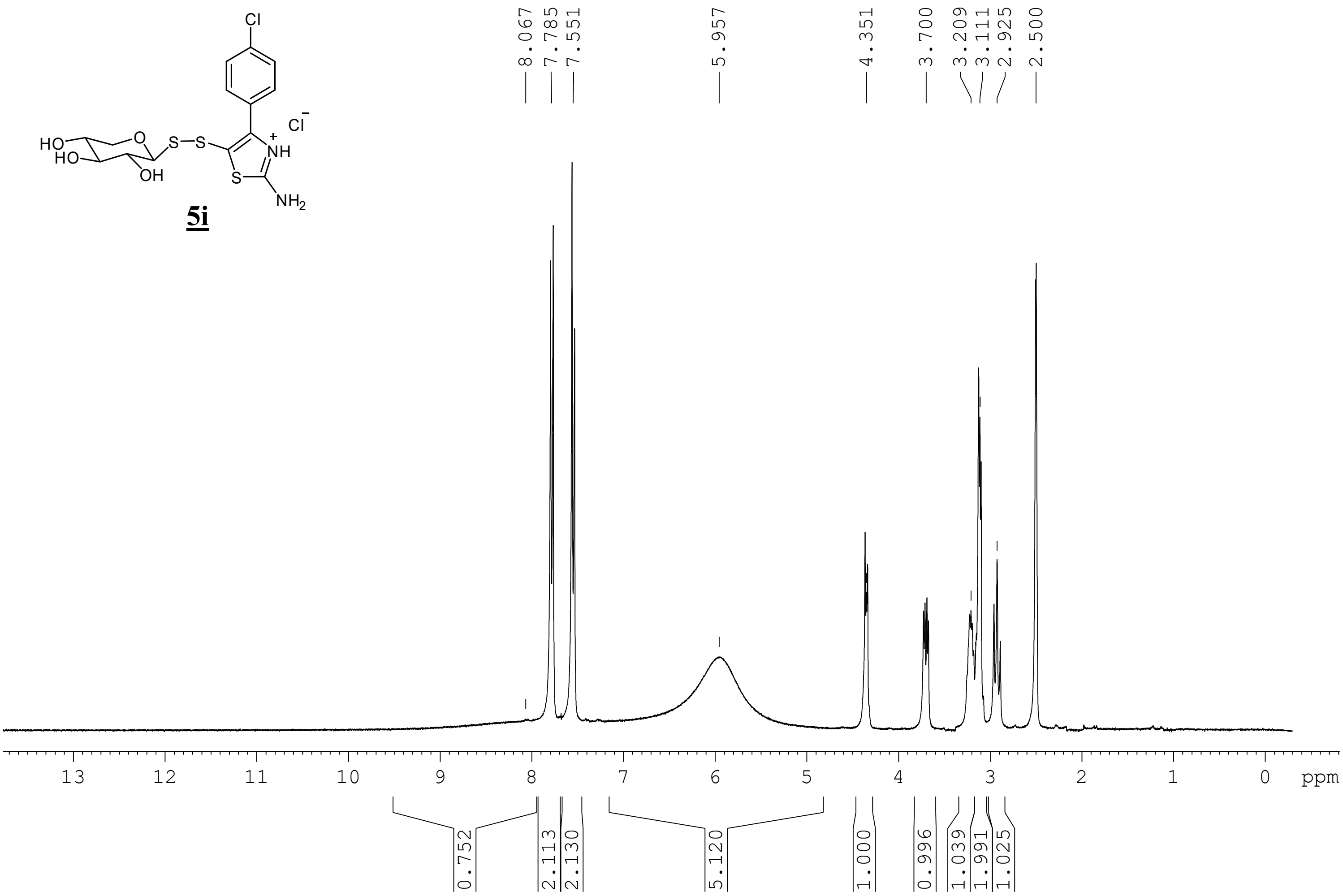
Cl
Cl⁻
HO
HO
OH
S–S
S
NH
NH2
5i
8.067
7.785
7.551
5.957
4.351
3.700
3.209
3.111
2.925
2.500
13 12 11 10 9 8 7 6 5 4 3 2 1 0 ppm
0.752
2.113
2.130
5.120
1.000
0.996
1.039
1.991
1.025

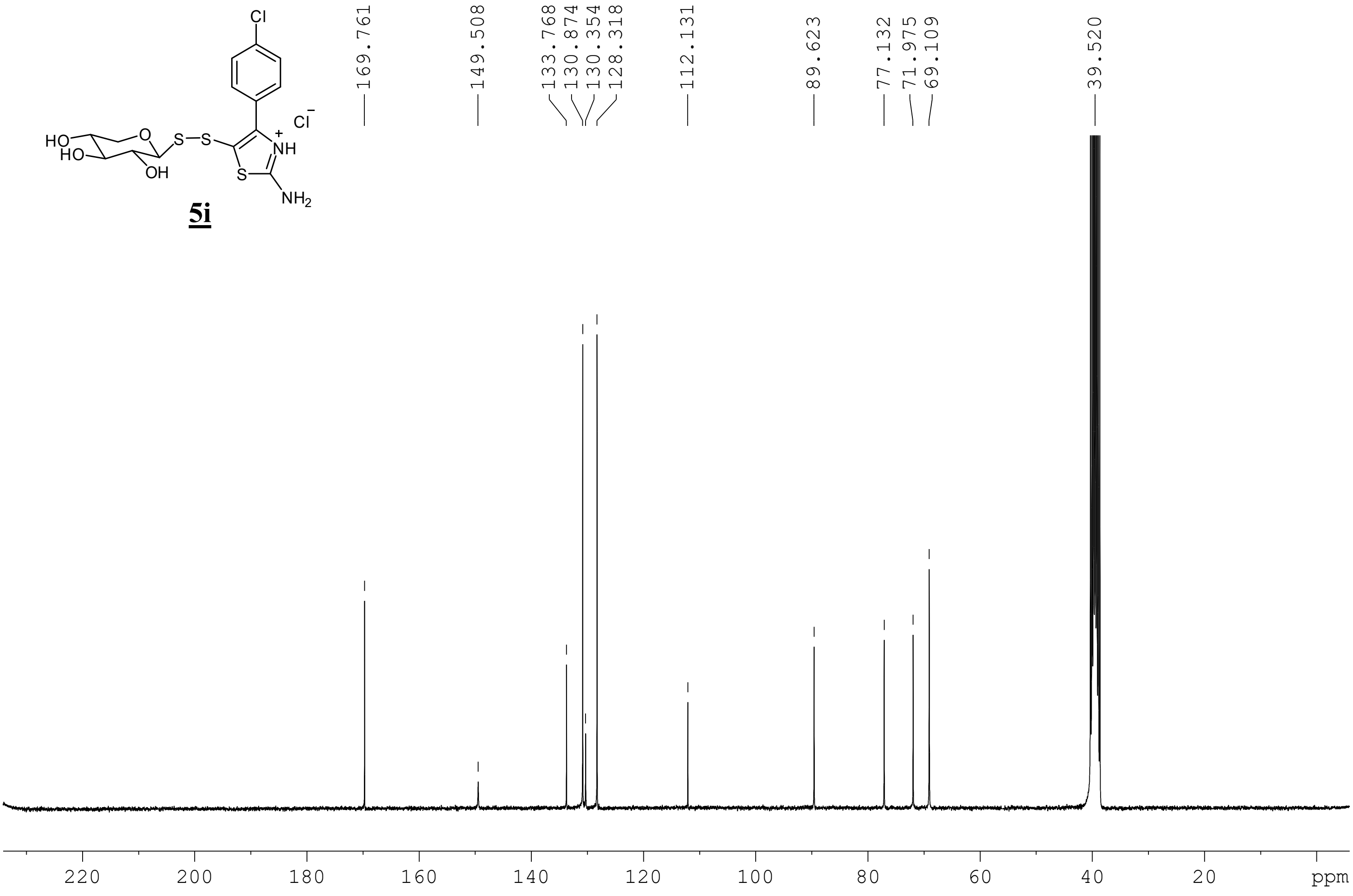
Cl
Cl⁻
HO
HO
OH
S–S
S
NH
NH2
5i
169.761
149.508
133.768
130.874
130.354
128.318
112.131
89.623
77.132
71.975
69.109
39.520
220 200 180 160 140 120 100 80 60 40 20 ppm

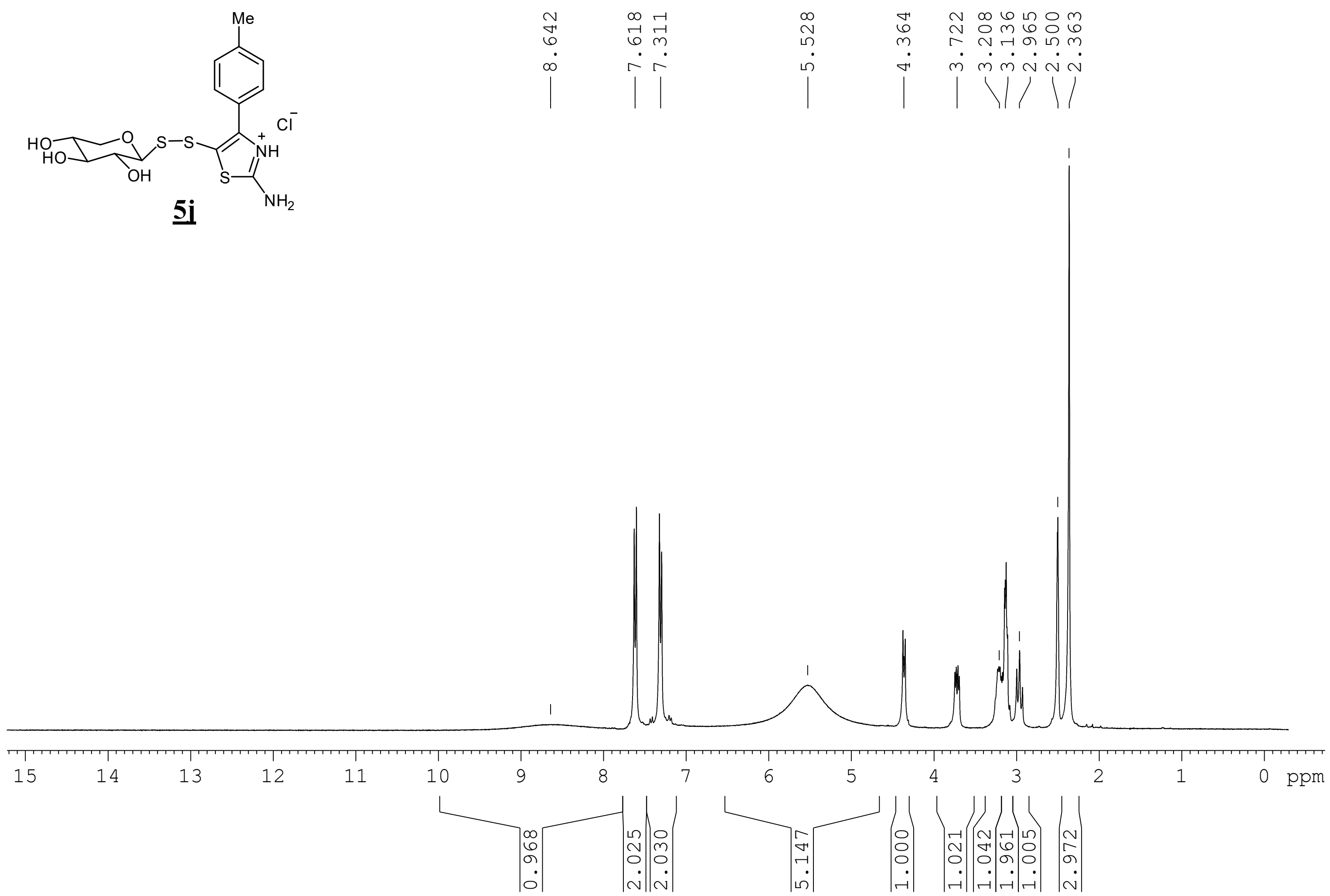
Me
Cl
HO
HO
O
S–S
OH
NH
S
NH2
5i
8.642
7.618
7.311
5.528
4.364
3.722
3.208
3.136
2.965
2.500
2.363
0.968
2.025
2.030
5.147
1.000
1.021
1.042
1.961
1.005
2.972
15 14 13 12 11 10 9 8 7 6 5 4 3 2 1 0 ppm

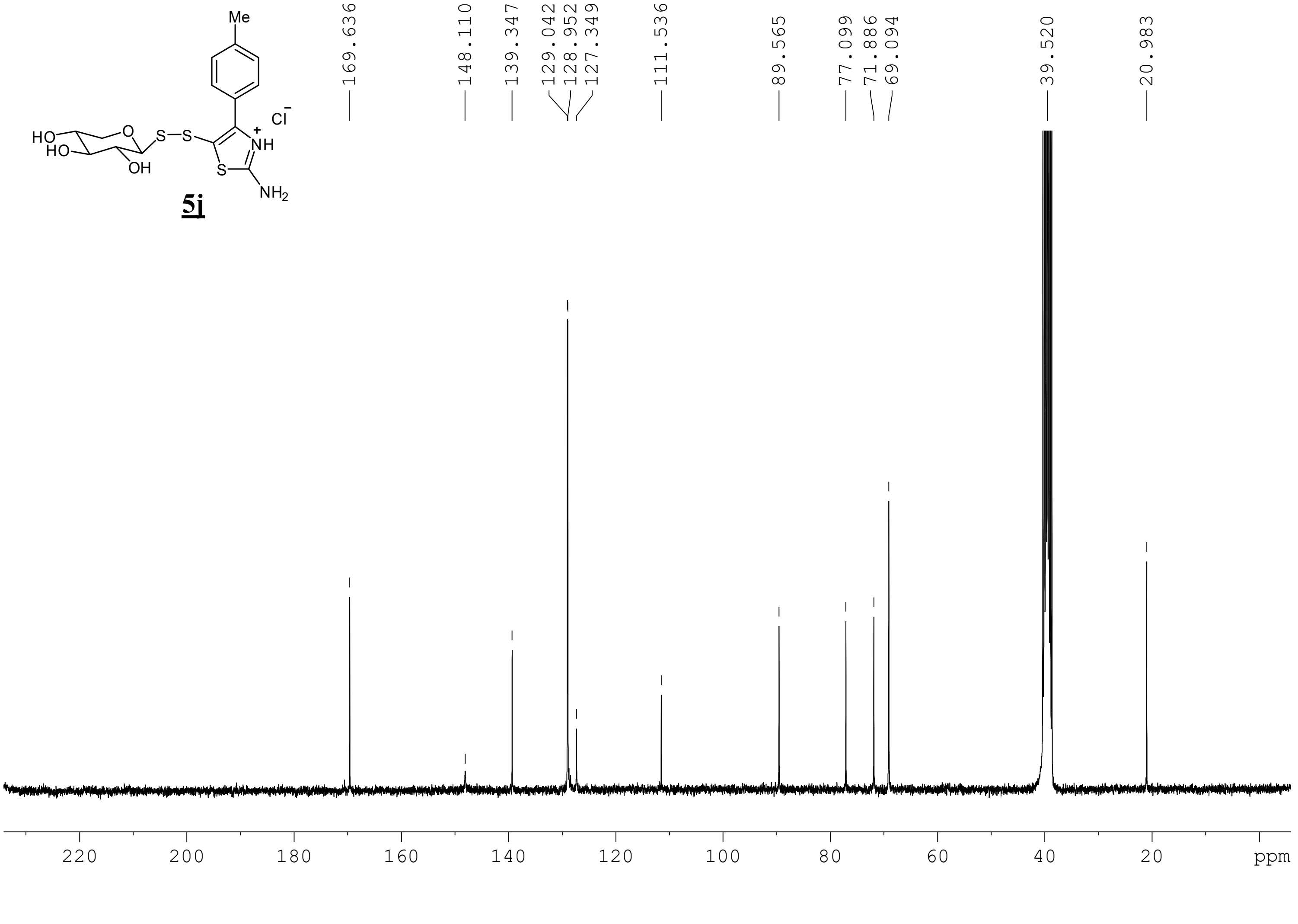
Me
Cl
HO
HO
O
S–S
OH
NH
S
NH2
5i
169.636
148.110
139.347
129.042
128.952
127.349
111.536
89.565
77.099
71.886
69.094
39.520
20.983
220 200 180 160 140 120 100 80 60 40 20 ppm
